# Multiple pathways of the actin-myosin cycle in energy transduction and the release of orthophosphate in muscle

**DOI:** 10.3389/fphys.2025.1664568

**Published:** 2025-11-04

**Authors:** Marco Caremani, Irene Pertici, Ilaria Morotti, Pasquale Bianco, Massimo Reconditi, Gabriella Piazzesi, Vincenzo Lombardi, Marco Linari

**Affiliations:** ^1^ PhysioLab, University of Florence, Florence, Italy; ^2^ Department of Biology, University of Florence, Florence, Italy; ^3^ Department of Experimental and Clinical Medicine, University of Florence, Florence, Italy

**Keywords:** chemo-mechanical coupling, orthophosphate release, energy transduction by myosin, actin–myosin cycle, unconventional actin–myosin ATPase cycle, myosin working stroke kinetics

## Abstract

In the striated muscle, the molecular motor myosin II functions in two bipolar arrays in each thick filament, converting chemical energy into steady force and shortening by cyclic ATP-driven interactions with nearby actin filaments. The fundamental steps in energy transduction are the working stroke, an inter-domain tilting of the lever arm about the actin-attached catalytic domain, generating up to ∼5 pN force or ∼10 nm of filament sliding, and the release of the ATP hydrolysis product orthophosphate (Pi) from the nucleotide-binding site, which is associated with a large free energy release. The two events are not simultaneous, as first demonstrated by the force response to a stepwise change in [Pi] (the Pi transient), showing the saturation kinetics characteristic of a two-step reaction. However, while high-resolution crystal structures of the myosin motor suggest that Pi release precedes the working stroke, *in vitro* functional studies indicate that it follows the working stroke. High-resolution sarcomere-level mechanics applied to single muscle fibers, allowing myosin motor synchronization by step perturbations in length or load, revealed that the kinetics of the working stroke is independent of [Pi] and depends only on the load. Moreover, this approach highlights the need for two unconventional pathways of the chemo-mechanical cycle: an early detachment of the force-generating motors and the possibility for attached motors to slip to the next actin monomer farther from the sarcomere center during shortening. Transient and steady-state responses to stepwise changes in load or [Pi] can be fitted with a structurally and biochemically explicit model in which the Pi release step is orthogonal to the progression of the working stroke. Model simulations indicate that the rate of Pi release depends on motor conformation, which resolves longstanding unanswered questions such as the dependence of Pi transient kinetics on the final level of [Pi] under any load and clarifies the issue of the relative timing between the working stroke and Pi release: at high loads, Pi release precedes the execution of the working stroke, while at low loads, the working stroke state transitions are fast enough to occur with Pi still bound to the catalytic site.

## Introduction

**FIGURE 1:**
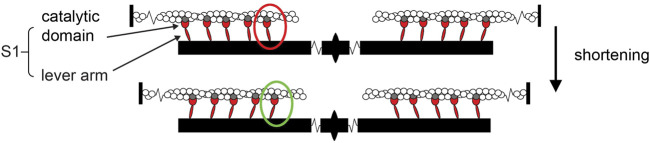
Cartoon representing the sarcomere and its shortening induced by the working stroke of myosin motors. Reciprocal sliding between the actin filaments (white), originating from the Z-line at the sarcomere extremities, and the myosin filaments (black), bound to the M-line at the sarcomere center and carrying two bipolar arrays of myosin motors (represented by their motor domains or sub-fragment 1 S1, red; all actin-attached for simplicity), is powered by the working stroke of the actin-attached myosin motor (red to green oval). The working stroke consists of inter-domain tilting of the lever arm about the catalytic domain, which remains firmly attached to the actin site (gray).

Force and shortening are generated in the sarcomere, the structural unit of striated muscle, by cyclical, ATP-driven interactions between myosin motors, which extend in two bipolar arrays from the center of the thick filament, and the overlapping thin actin-containing filaments that originate from the sarcomere extremities (Figure 1).

In each interaction, the free energy of the hydrolysis of ATP in the catalytic site of the myosin motor is converted into mechanical work through a structural change (the working stroke) that, according to the crystallographic model (Rayment et al., 1993a; Rayment et al., 1993b), consists of an inter-domain tilting of the lever arm about the catalytic domain firmly attached to the actin site. The working stroke is associated with the sequential release of the hydrolysis products orthophosphate (Pi) and ADP. Calorimetric and protein solution studies have shown that a large enthalpy change is related to Pi release from the quaternary complex actin–myosin-ADP-Pi (AMADPPi), suggesting that the Pi dissociation step is the chemical transition associated with the mechanical working stroke (White and Taylor, 1976; Hibberd et al., 1985; Hibberd and Trentham, 1986).

**SCHEME 1:**
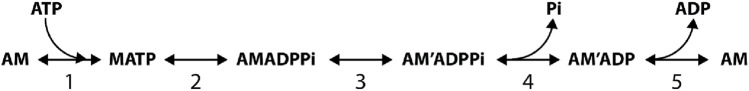
5 step reaction scheme from Dantzig et al., 1992.

Photo-liberation of caged Pi in skinned muscle fibers showed that the force during an isometric contraction is reduced by a stepwise increase in [Pi] with a rate that increases asymptotically with [Pi] (Dantzig et al., 1992; Millar and Homsher, 1992; Walker et al., 1992; Homsher et al., 1997), implying that, according to the minimum five-step reaction of Scheme 1, the Pi release (step 4) follows the force-generating transition from a weak AMADPPi to a strong AM'ADPPi state (step 3).

In Reaction Scheme 1, which summarizes the results from solution protein studies and caged compound experiments in skinned fibers, ATP binding to the actomyosin (AM) complex, after the release of the hydrolysis products and the execution of the working stroke, promotes rapid dissociation of myosin from actin (step 1), which is followed by the recovery stroke (the reversal of the working stroke) and ATP hydrolysis (step 2). The detached myosin with the hydrolysis products is in rapid equilibrium with the weakly bound AMADPPi state. The closure of the actin-binding cleft of the myosin catalytic domain (Geeves and Holmes, 2005) forms the strongly attached, stiffness-generating AM'ADPPi state, which is allosterically associated with the structural change that leads to tilting of the lever arm responsible for force generation and filament sliding (step 3). Thus, according to the simplified Scheme 1, step 3 is the combination of two processes, namely, the formation of a strongly bound motor and the generation of force, with kinetics limited by the much slower attachment process. This definition of step 3 is in agreement with the fast kinetics of force generation, as estimated from the quick force recovery following a step release (Huxley and Simmons, 1971; Ford et al., 1977), and it is supported by the evidence that the force increases in proportion to the number of myosin motors during isometric force development by a muscle fiber (Brunello et al., 2006; Caremani et al., 2008). Pi is released without any further contribution to force (step 4, Dantzig et al., 1992). Thus, the sum of the fractional occupancies of the AM'ADPPi and AM'ADP states constitutes the fraction (*f*) of actin-attached motors that contribute equally to the force and stiffness. The subsequent ADP release (step 5) occurs at a rate that, in the isometric contraction, is low (Nyitrai and Geeves, 2004; Sleep et al., 2005; West et al., 2005). According to Scheme 1, the development of isometric force is rate-limited by both the ATP hydrolysis step (step 2) and the attachment of motors (step 3), while the steady-state flux through the whole cycle (the rate of ATP hydrolysis) is limited by the rate of the ADP release (step 5). ADP release is conformation-dependent and becomes relatively fast only when attached motors preferentially populate the final state of the working stroke, such as during shortening at low load (Nyitrai and Geeves, 2004; Caremani et al., 2015). Thus, *in vitro* and *in situ* kinetic studies support Scheme 1 as the unique path through which myosin–actin interaction in muscle hydrolyzes ATP and produces force and power.

This conclusion, however, is questioned on the basis of several pieces of challenging evidence, which are discussed in detail in the next sections. First, most crystallographic models of the myosin motor domain (sub-fragment 1, S1) (Smith and Rayment, 1996; Houdusse et al., 2000; Geeves and Holmes, 2005; Llinas et al., 2015) support the alternative view that Pi is released prior to the working stroke (see also Robert-Paganin et al., 2020; Moretto et al., 2022; Mansson et al., 2023; Rassier and Mansson, 2025). Second, the finding that an increase in [Pi] reduces the isometric force of the Ca^2+^-activated fibers more than the ATPase rate (Cooke and Pate, 1985; Caremani et al., 2008) suggests that an increase in [Pi] increases the probability of an alternative pathway of the chemo-mechanical cycle (Linari et al., 2010; Debold et al., 2013), with premature detachment of motors from a strongly bound force-generating state, followed by the release of the hydrolysis products. Third, the attachment–detachment kinetics that fits the rate of isometric force generation is too slow in relation to the maximum power developed during steady shortening, which suggests that during shortening, an attached motor could complete its ATPase cycle by slipping to the next actin monomer farther from the sarcomere center (Lombardi et al., 1992; Piazzesi and Lombardi, 1995; Caremani et al., 2013; Pertici et al., 2023).

## Relation between the release of Pi and the working stroke

**FIGURE 2:**
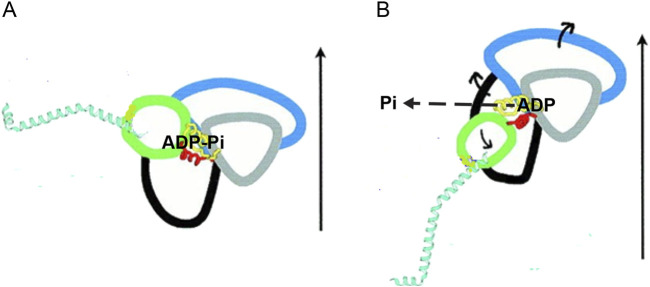
Schematic drawing of the subdomains within the myosin catalytic domain and their reciprocal structural changes accompanying the working stroke transition. The four subdomains identified by different colors are the N-terminal (black), the upper 50-kDa subdomain (blue), the lower 50-kDa subdomain (gray), and the converter (green). Two (yellow and red) of the structural elements joining the subdomains are visible. The α-helix constituting the lever arm is cyan. The structures are obtained from the scallop myosin: **(A)** S1 complexed with VO_4_^3-^, representing the pre-working stroke state. **(B)** Nucleotide-free S1, representing the end-of-stroke state. The vertical arrows indicate the approximate direction of the actin filament axis. The orientation of the two structures is defined by conserving the orientation of the lower 50-kDa relative to actin. The release of Pi is indicated with the dashed arrow. The exit direction is an open question, and more recent evidence (Llinas et al., 2015) indicates that it is opposite (backdoor) to what is shown here. The direction of the movement of the subdomains in the transition between the two states is shown by the short continuous arrows. The ∼70° change in the orientation of the converter is turned into ∼11 nm axial movement by the length of the lever arm (modified from Figure 2B by Houdusse et al. (2000). Copyright (2000) National Academy of Sciences, United States).

Crystallographic studies (Smith and Rayment, 1996; Houdusse et al., 2000; Geeves and Holmes, 2005; Llinas et al., 2015) have the unique ability to provide details of the inter-domain movements within the motor domain (S1, Figure 1) that associate the closure of the actin-binding cleft, which is responsible for the formation of the strongly-bound motor, to the catalytic site and then to the converter that controls the orientation of the lever arm (Figure 2). At the catalytic site, modifications in the orientation of the relevant structural elements (P-loop, switch 1, and switch 2) that join the subdomains promote both a decrease in affinity for orthophosphate and a chain of changes in neighboring residues, culminating in a tilt in the converter.

In contrast to this view, Förster resonance energy transfer (FRET) which, unlike crystallography, allows for the direct recording of structural dynamics within the myosin motor, indicates that the working stroke precedes Pi release (Muretta et al., 2015). Similar conclusions have been reached with single-molecule mechanics using a three-bead assay system (Takagi et al., 2004), which were later confirmed with an enhanced time resolution obtained by eliminating the effect of the trap compliance by imposing resistive loads from the very beginning of the interaction (Capitanio et al., 2012; Woody et al., 2019). A critical issue in all the above *in vitro* mechanical studies is that the loading condition under which the myosin–actin interaction occurs is far from reproducing the physiological load range, while the phenomena under investigation, the working stroke and the release of Pi, have individual unknown load sensitivities as far as their extent and kinetics are concerned. In particular, it must be considered that FRET measurements were achieved in unloaded conditions, and thus, the fractional occupancy of attached motors derived from this study is biased to the state at the end of the working stroke. On the other hand single-molecule measurements showed a working stroke rate that increases and a lifetime of actin-attached motors that decreases with the increase of the resisting load, opposite to the relations expected from the bulk of *in situ* mechanical and energetic data (Hill, 1938; Huxley and Simmons, 1971; Reconditi et al., 2004; Pertici et al., 2023).

**FIGURE 3:**
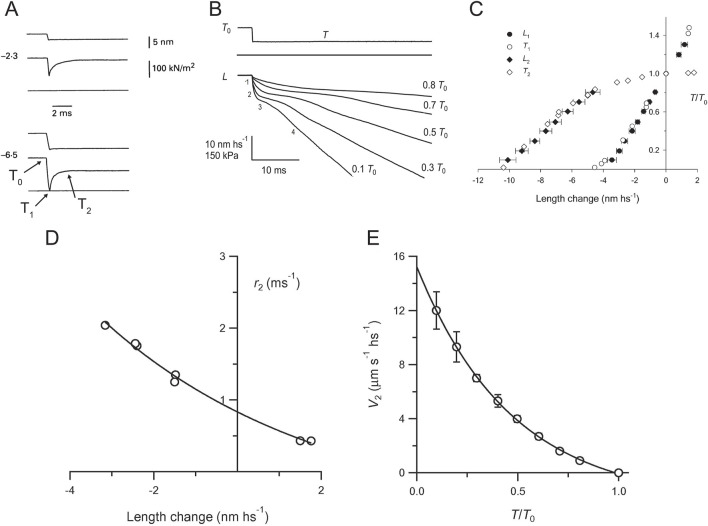
Force and velocity transients. **(A)** Early components of force transients (middle trace in each panel) elicited by a step release (upper trace) superimposed on the steady force of the isometric contraction (*T*_0_) of a single fiber from frog skeletal muscle. Release amplitude per half-sarcomere (hs), 2.3 nm (upper panel) and 6.5 nm (lower panel). The lower horizontal line is the baseline for force. **(B)** Isotonic velocity transients following steps in force superimposed on the isometric force (*T*_0_) of a single fiber from frog skeletal muscle. Upper trace, stepwise decrease to a force *T* = 0.5 *T*_0_; middle trace, force baseline; lower traces, change in half-sarcomere length (*L*) corresponding to steps to the forces indicated by the figures on the right. The figures on the left of the 0.1 *T*_0_ trace show the phases of the transient. Reproduced from Figure 1 of Piazzesi et al. (2002) with permission. **(C)** Comparison of *L*_1_ (filled circles) and *L*_2_ (filled diamonds) relations from velocity transients with *T*_1_ (open circles) and *T*_2_ (open diamonds) relations from force transients. Reproduced from Figure 4 of Piazzesi et al. (2002) with permission. **(D)** Rate of early force recovery (*r*_2_) versus step amplitude. The line is obtained by fitting the *r*_2_ data points with an exponential. Reproduced from Figure 3C of Piazzesi and Lombardi (1995). **(E)** Relation between the initial shortening velocity of phase 2 (*V*_2_) and force (*T*/*T*_0_). The line is an exponential fit to the data. Reproduced from Figure 3A of Piazzesi et al. (2002).

In our experience, the most reliable approach for clarifying the issue of the relative timing and load-dependence of the working stroke and the Pi release is the sarcomere-level mechanics with nanometer-microsecond resolution achieved by integrating fast force transducers (Huxley and Lombardi, 1980) with the striation follower (Huxley et al., 1981), which was first developed for intact fibers isolated from frog skeletal muscle (Lombardi and Piazzesi, 1990) and then applied to single skinned fibers of the mammalian skeletal muscle (Linari et al., 2007; Caremani et al., 2013) (Supplementary Figure 1A). The striation follower is an optoelectronic apparatus that enables the measurement and control of half-sarcomere (hs) length changes in a selected population of sarcomeres, with sub-nanometer precision and 2-μs time resolution. As shown in Figure 3A and Supplementary Figure 1B, a stepwise reduction of half-sarcomere length by a few nanometers (complete in ∼100 μs) superimposed on the isometric contraction elicits a force transient composed of a force change simultaneous with the length step (phase 1) to a value T_1_, which depends on elastic properties of the half-sarcomere, followed by a force recovery due to the active properties of the myosin motor. The earliest component is a rapid force recovery (phase 2), which represents the mechanical manifestation of the working stroke of attached motors synchronized by the step release. For releases larger than 6 nm per hs, the force recovered in phase 2 is only a fraction of the force decrease during the elastic response and is followed by a pause in force recovery (phase 3) that precedes the final recovery to the original isometric force *T*_0_ (phase 4). Phase 3 results from the synchronous attainment of the end of the working stroke by attached motors and their accelerated detachment, whereas phase 4 is explained by the isometric attachment–detachment kinetics. Phase 2 recovery holds the information used for developing a model of the working stroke (Huxley and Simmons, 1971; Ford et al., 1977): the maximum extent of filament sliding for which attached motors can maintain force (estimated by the abscissa intercept of the *T*_2_ relation) was ∼11 nm (Figure 3C), which is in agreement with the crystallographic model of the working stroke proposed 20 years later (Rayment et al., 1993a). The speed of phase 2 force recovery, *r*_2_, measured as the reciprocal of the time to recover from *T*_1_ to *T*_1_ + 0.63*(*T*_2_-*T*_1_), is larger for release than for stretch and increases with the release size (Figure 3D). This dependence of *r*_*2*_ on the step size and direction excludes the response of a passive viscoelasticity and holds the constraints for the kinetic model of force generation (Huxley and Simmons, 1971).

**FIGURE 4:**
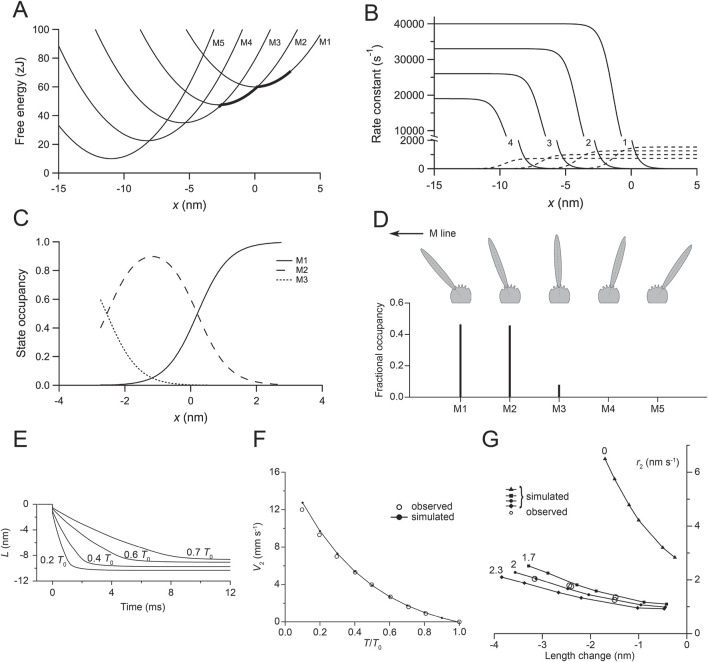
Relevant parameters of the kinetic model of the working stroke. **(A)** Free energy diagram of the myosin motor states (M_1_–M_5_) as a function of the relative position (*x*) between the myosin motor and the actin monomer. *x* is set to 0 for the minimum free energy of an attached motor in the state M_1_. The free energy minima of the various states are separated by a distance *z* (the step size) = 2.75 nm so that the working stroke of 11 nm is preserved with a number of structural transitions = 4. The thick line marks the axial distribution of motors during the isometric contraction. **(B)** Functions expressing the *x*-dependence of the forward (continuous lines) and backward (dashed lines) transitions. The figures indicate the order of the transition. **(C)** Fractional occupancy of the various states as a function of *x* during isometric contraction. **(D)** Sketch of the conformation of the five states (M_1_–M_5_) of the myosin motor characterized by orientations of the lever arm separated by angles corresponding to an axial displacement of 2.75 nm of the tip of the lever under zero force. The lower panel shows the fractional occupancy of the different states in isometric contraction. **(E)** Simulated velocity transients following force decreases to the values indicated by the figures close to the traces. **(F)** Filled circles and continuous line: simulated *V*_2_-*T/T*_0_ relation; open circles: experimental data from Figure 3E. **(G)** Filled symbols and lines: simulated relations between *r*_2_ and the size of the step release in the absence of filament compliance (triangles) and in the presence of 1.7 nm/T_0_ (squares), 2 nm/T_0_ (filled circles), and 2.3 nm/T_0_ (diamonds) filament compliance. Open circles are experimental data from Figure 3D. Panels A–C, reproduced from Figure A1 of Piazzesi et al. (2014); panel D, reproduced from Figure 4 of Fusi et al. (2014); panels E and F, reproduced from Figure 4 of Piazzesi et al. (2014); panel G, reproduced from Figure 5D of Piazzesi et al. (2014).

However, the presence of a significant compliance in the myofilaments, functionally in series with that of the motor array in each half-sarcomere (Huxley et al., 1994; Wakabayashi et al., 1994), complicates the use of the rate of quick force recovery *r*_2_ as the constraint for modeling the working stroke kinetics (Linari et al., 2009; Piazzesi et al., 2014). The rationale is illustrated with a simplified mechanical model of the half-sarcomere (Supplementary Figure 2), in which the distributed compliance of actin and myosin filaments is represented by an elastic element in series with the array of myosin motors. Under these conditions, force recovery occurs while motors move in the shortening direction to increase stress in the filaments, and its rate *r*_2_ decreases in proportion to filament compliance. The availability of a capacitance force transducer with a resonant frequency of 50 kHz (Huxley and Lombardi, 1980) and, thus, an adequately fast force clamp enabled an alternative protocol in which the velocity transient was recorded following stepwise decreases in force superimposed on the isometric steady force (Figure 3B; Piazzesi et al., 2002). The elastic shortening in phase 1 (*L*_1_) simultaneous with the force step is followed by a rapid phase-2 shortening (due to the synchronization of the working stroke in the attached motors) with an extent (*L*_2_ - *L*_1_, where *L*_2_ is the hs shortening attained at the end of phase 2) that is larger and attained earlier at lower force. *L*_2_ (the sum of the elastic strain and the working stroke) for a decrease in force to zero coincides with the abscissa intercept of the *T*_2_ relation (Figure 3C). Phase-2 shortening is followed by a pause (phase 3, due to synchronized detachment of motors) and then a steady shortening (phase 4) at a lower velocity that is characteristic of the detachment–attachment kinetics underpinning the force–velocity relation of the contracting muscle. The phases that follow the elastic (phase 1) response occur under force clamp and, thus, are not affected by the compliance of myofilaments. Under this condition, the dependence on force of the speed *V*_2_ estimated by the tangent to the initial part of phase-2 shortening (Figure 3B) directly measures the load dependence of the speed of the working stroke (Figure 3E), providing the constraints for a mechanical–kinetic model of the myosin working stroke *in situ* (Figures 4A–C).

For simplicity, the model considers only the mechanical and kinetic properties of the attached myosin motors that determine phase 2 of the responses to length and force steps; thus, the kinetic scheme is based on Huxley–Simmons 1971 model (Huxley and Simmons, 1971) and does not include further steps of the actin–myosin interaction, such as the attachment and detachment of motors. In this study, we provide a synthetic description of the model, which is described in detail by Piazzesi et al. (2014). The stiffness of the myosin motor is 3 pN/nm, and this sets the slope of the parabolas representing the free energy profile of attached states and, thus, the size (2.75 nm) and number (4) of the force-generating steps necessary to fit the transition and equilibrium kinetics of the working stroke. The rate functions of the transitions between the five structural states marking the progression of the working stroke (Figure 4D) were selected by the model simulation to achieve the best fit of the phase-2 velocity transient and its dependence on load (Figures 4E, F). Then, the same kinetic scheme was applied to simulate the relation between the rate of phase-2 force transient (*r*_2_) and the size of the step release (Figure 4G, filled triangles). The simulated relation was shifted upward with respect to the observed relation (open circles), with the simulated *r*_2_ already four times higher for 1.5 nm release. The difference between the observed and simulated relations was the consequence of neglecting the depressant effect of filament compliance on the rate of force generation by the motor array in the model. Assuming that the filament compliance was the only adjustable parameter, it was found that the same kinetic scheme can fit the observed relation when an equivalent filament compliance of ∼2 nm/*T*_0_ per hs is assumed (filled circles), a value in fairly good agreement with the filament compliance estimated with mechanical and X-ray diffraction experiments on the same preparation (Brunello et al., 2014).

The simplified kinetic scheme does not imply detachment/attachment; thus, the simulation of the velocity transients ends with the equilibrium distribution of attached states at each force, which underpins working stroke amplitude that reduces with the load, mostly due to the reduced contribution of the recoil of elastic elements (Figure 4E). The reduction in the observed working stroke amplitude with increase in the load is larger (Figure 3B) because the completion of the working stroke is progressively truncated by the ensuing synchronized detachment in phase 3.

**FIGURE 5:**
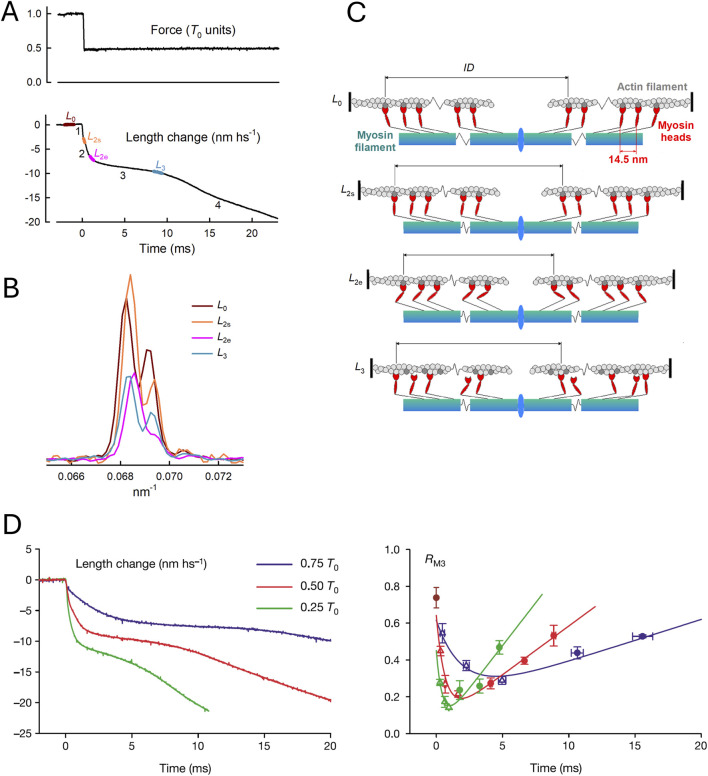
Motion of myosin motors during the velocity transient. **(A)** Force change normalized by isometric force *T*_0_ and length change in nm/hs. **(B)** Axial X-ray intensity distribution in the region of the M_3_ reflection recorded at the BioCAT beamline of the APS Synchrotron (Argonne, United States). Colors denote X-ray exposure periods, as shown in **(A)** brown, *L*_0_; orange, *L*_2s_; magenta, *L*_2e_; and cyan, *L*_3_. **(C)** Structural organization of myosin motors in the muscle sarcomere at each X-ray exposure, as predicted by the M3 interference fine structure in **(B)**. **(A–C)** From Figure 1 of Reconditi et al. (2004). **(D)** Superimposed half-sarcomere shortenings (left panel) following force decreases to three different values (identified by the color code in the inset) and intensity ratio (*R*_M3_, right panel) of the higher over the lower angle component of the M3 intensity distribution. From Figure 2A, B of Reconditi et al. (2004).

These conclusions were straightforwardly confirmed in their structural counterpart, achieved by using the same mechanical protocol (Figure 5A) in combination with time-resolved X-ray diffraction from synchrotron light (Supplementary Figure 1) (Reconditi et al., 2004).

X-ray diffraction by itself lacks the phase information required to directly define the change in shape of the diffracting unit, but the size and direction of the movement can be recovered with sub-nanometer precision by exploiting the X-ray interference between the two bipolar arrays of motors in each sarcomere. Following the decrease in force, the changes in the interference fine structure of the so-called M3 reflection originating from the 14.5-nm axial periodicity of myosin motors (Figure 5B) indicate the movement of attached motors toward the center of the sarcomere in phase 2, coherent with the execution of the working stroke, and their movement away from the center at the onset of phase 3, associated with motor detachment at the end of the working stroke followed by re-attachment farther from the sarcomere center (Figure 5C). The structural data (Figure 5D, right panel) match the striation follower signal (left panel), indicating a larger and faster working stroke with decreasing load.

**FIGURE 6:**
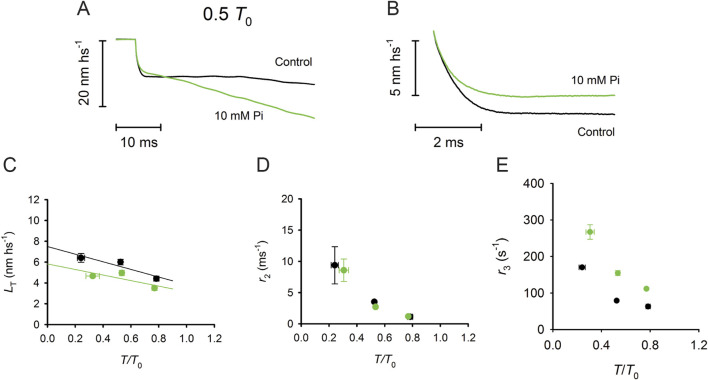
Effect of [P_i_] on the isotonic velocity transient. **(A)** Superimposed isotonic velocity transients following a force step to 0.5 *T*_0_ in control (black) and 10 mM P_i_ (green). **(B)** *S*uperimposed phase 2 of the velocity transients in control (black) and in 10 mM P_i_ (green), which were obtained after subtracting phase-1 and phase-3 shortenings. **(C)** Relation between the shortening accounted for by the working stroke (*L*_T_) and the force expressed in relative units *T/T*_0_ in control (black) and 10 mM P_i_ (green). **(D)** Dependence of the rate constant of the working stroke (*r*_2_) on *T*/*T*_0_ in control (black) and 10 mM P_i_ (green). **(E)** Dependence of the rate of phase 3 (*r*_3_) on *T*/*T*_0_ in control (black) and 10 mM P_i_ (green). Reproduced from Figures 2, 3 of Caremani et al. (2013) with permission.

A reliable method for *in situ* recording of the working stroke is the prerequisite for investigating its sensitivity to [Pi]. By applying the striation follower technology and the fast force clamp protocols to skinned fibers from rabbit psoas (Caremani et al., 2013), it has been shown that increase in [Pi] to 10 mM (i) reduces the isometric force by 35%, without a significant effect on the unloaded shortening velocity (*V*_0_) (see also Cooke et al., 1988), (ii) does not affect the rate constant of the working stroke, as measured by the speed of phase-2 shortening, and (iii) shortens phase-3 pause and accelerates the subsequent transition to the final steady shortening velocity (Figure 6).

Most of the literature, in contrast to the above conclusion, reports a direct effect of Pi on the force generation process. This finding can be explained considering that those studies lack the resolution to discriminate between motor attachment and force generation (for instance, the process 2πb identified with sinusoidal analysis by Kawai and Halvorson, 1991). A study on the effect of [Pi] on the rate of the working stroke, which was determined in the same preparation using the quick force recovery elicited by length steps (Ranatunga et al., 2002), produced contradictory results: increasing [Pi] did not affect the rate of quick force recovery following a step release, whereas it increased the rate following a step stretch, mainly at the expense of its latter component. These results, however, can be justified by taking into account that in the quick force recovery following a step stretch, there is a contamination of the reversal of the working stroke by rapid detachment–attachment. With double-step protocols in single frog muscle fibers, it has been demonstrated that (*i*) the quick force recovery from a step release up to 6 nm per hs is complete within 2 ms and is almost fully accounted for by the synchronous execution of the working stroke because the detachment–attachment responsible for the rapid repriming of the working stroke is three times slower (Lombardi et al., 1992); (*ii*) the recovery from a stretch of 4 nm (a size comparable to that of Ranatunga et al., 2002), instead, takes ∼20 ms, and only 1/3 of it is explained by the synchronous reversal of the working stroke while the remaining part is due to the ensuing detachment–attachment that, in the force transient following a stretch, merges with the reversal within phase 2 (Piazzesi et al., 1997). Thus, the sensitivity to [Pi] of the later part of the recovery from a stretch is explained by the Pi-dependent acceleration of detachment in phase 3 of the velocity transient (Caremani et al., 2013).

In summary, the analysis of the velocity transients shows that the rate of the working stroke is independent of [Pi] and solely depends on the load and that the increase in [Pi] accelerates the phase-3 detachment–attachment (Caremani et al., 2013). These conclusions constrain the modeling of the chemically and structurally explicit cycle implemented from Reaction Scheme 1 (Reaction Scheme 4 presented later) to simulate both transient (phases 2 and 3) and steady-state (phase 4) shortening responses following the decrease in force below *T*_0_ (Caremani et al., 2013), which is in agreement with the original idea of Huxley and Simmons (1971) that the progression through the working stroke is controlled by rate functions that solely depend on the strain under which the transition from a lower to a higher force-generating state occurs. At the same time, the release of Pi from the catalytic site of the myosin motor can occur at any stage of the structural transitions. In this way, the release of Pi is an orthogonal process to the working stroke, and its rate does not directly depend on the motor strain, which is compelling for a chemical step. At the same time, as detailed in the section describing the properties of Reaction Scheme 4, the rate of Pi release can be made conformation-dependent, in which case it increases as the motor progresses through the working stroke.

## Evidence for premature motor detachment from a strongly bound force-generating state induced by an increase in [Pi]

**FIGURE 7:**
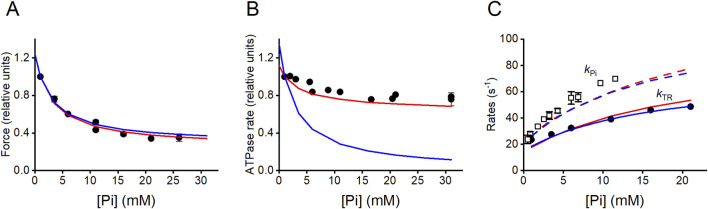
Effects of [Pi] on the isometric force, ATPase rate, and rate of force change during initial force increase and following a Pi jump. **(A)** Pi-dependence of isometric force (relative to the force in the control solution, 1 mM Pi). Filled circles, data from Figures 1F, 6A of Caremani et al. (2008). Model predictions: blue line, Scheme 1; red line, Scheme 2. **(B)** Pi-dependence of the ATPase rate: filled circles, pooled data from Bowater and Sleep (1988), Potma et al. (1995) and Potma and Stienen (1996). Model predictions: blue line, Scheme 1; red line, Scheme 2. Values relative to those in the control solution. **(C)** Pi-dependences of *k*_TR_ (the rate of force redevelopment from a large release bringing the force to 0) and *k*_Pi_ (the rate of force decrease following a Pi jump). Filled circles, observed *k*_TR_ relation from Figure 6C of Caremani et al. (2008). Model prediction: blue continuous line, Scheme 1; red continuous line, Scheme 2. Open squares, observed *k*_Pi_ relation from Figure 6 of Dantzig et al. (1992). Model prediction: blue dashed line, Scheme 1; red dashed line, Scheme 2. Modified from Linari et al. (2010).

Addition of Pi to the solution induces a decrease in the Ca^2+^-activated steady isometric force (*T*_0_) of a skinned fiber (Figure 7A) (Cooke and Pate, 1985; Kawai and Halvorson, 1991; Dantzig et al., 1992; Caremani et al., 2008) and, to a lesser extent, a decrease in the isometric ATPase rate (Figure 7B) (Webb et al., 1986; Bowater and Sleep, 1988; Cooke et al., 1988; Potma et al., 1995; Potma and Stienen, 1996). In 10 mM Pi, *T*_0_ is reduced to ½ the value in control solution (with 0.7 mM–1 mM of contaminating Pi Pate and Cooke, 1989a; Millar and Homsher, 1990), while the ATPase rate is reduced by less than 20%.

**SCHEME 2:**
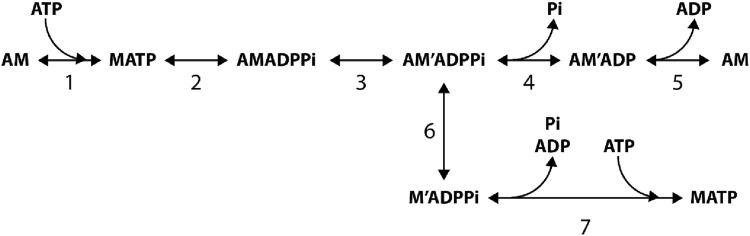
5 step reaction scheme integrated by a branched pathway accounting for the detachment from a strong bound AM'ADPPi state followed by release of hydrolysis products.

In terms of a conventional chemo-mechanical cycle in which Pi release occurs with the working stroke (Pate and Cooke, 1989a; Pate and Cooke, 1989b), the apparent contradiction was explained by hypothesizing that an increase in [Pi] shifts attached motors toward a state generating lower force without any effects on the ATP turnover. However, this idea was contradicted by evidence that the Pi-dependent reduction in force is accompanied by a proportional reduction in the number of attached motors (Caremani et al., 2008), a finding anticipated by work using sinusoidal analysis (Kawai and Halvorson, 1991), even though the conclusion in that case appeared questionable because of both the limited time resolution and the absence of analysis of the effects of filament compliance. A Pi-dependent reduction in the number of attached motors in isometric contraction without a comparable reduction in the ATP turnover indicates that the flux of myosin motors through the attachment/force-generating step (step 3 in Reaction Scheme 1) is larger than the flux through the conventional cycle estimated through steps 5 and 1, demonstrating that the conventional model cannot explain the reduced effect of [Pi] on the ATPase rate with respect to that on the force. The problem is solved by assuming a branched pathway that allows the myosin in the AM'ADPPi force-generating state to detach from actin before the release of ADP and Pi and then quickly release the hydrolysis products through an unconventional cycle (see Reaction Scheme 2; Linari et al., 2010). This conclusion was later supported by the results and modeling of the effect of Pi on the *in vitro* mechanics of a mini-ensemble of myosin motors (Debold et al., 2013). Notably, to be effective, the release of hydrolysis products from the AM'ADPPi state must be assumed irreversible; so, for the mass action, an increase in Pi promotes only the flux through the AMADPPi state.

Notably, the branched pathway introduced in Reaction Scheme 2, while enhancing the ATP consumption at any [Pi] fitting the observed relation (red line in Figure 7B), maintains the property of Reaction Scheme 1. It fits the dependence on [Pi] of either the rate of isometric force development (*k*_TR_, Figure 7C; filled symbols, experiment; continuous blue line, Reaction Scheme 1; continuous red line Reaction Scheme 2) or the rate of force reduction elicited by a stepwise increase in [Pi] produced by photo-liberation of caged Pi during the isometric contraction (*k*_Pi_, Figure 7C; open symbols experiment; dashed blue line Reaction Scheme 1; dashed red line Reaction Scheme 2) equally well. This is because both the schemes assume that force generation and Pi release occur as a two-step process, with the first step (force generation, corresponding to step 3 in Reaction Scheme 1) and the second step (the release of Pi, corresponding to step 4 in Reaction Scheme 1) being sufficiently fast (>500/s) to be considered in rapid equilibrium, with a K ∼10 mM (Dantzig et al., 1992; Homsher et al., 1997).

### Pros and cons of Reaction Scheme 1 from Pi-transient experiments

The force decrease in response to an increase in [Pi] (Pi transient) imposed on Ca^2+^-activated fibers exhibits an amplitude *A*_Pi_ and a rate *k*_Pi_ that provide the fundamental constraints of the kinetics of the underlying reactions. Dantzig et al. (1992) found that the observed *k*_Pi_ can be restricted to a section of the whole cycle involving the two-step reaction:

$$k_{Pi}=k_{a}+k_{b}*\frac{\left[\text{Pi}\right]}{\left(K_{c}+\left[Pi\right]\right)},$$ (1)

where *k*_a_ and *k*_b_ are the forward and backward rate constants of step 3 in Reaction Scheme 1 and *K*_c_ is the equilibrium constant of Pi release. The process 2πb of sinusoidal analysis (Kawai and Halvorson, 1991) follows the same two-step reaction kinetics. Notably, the rate of isometric force development following a large release that reduces the force to 0, *k*_TR_, which involves the whole attachment–detachment cycle, shows a Pi-dependence with saturation kinetics (Figure 7C, filled circles) that holds the footprint of the two-step reaction of Equation 1.

**FIGURE 8:**
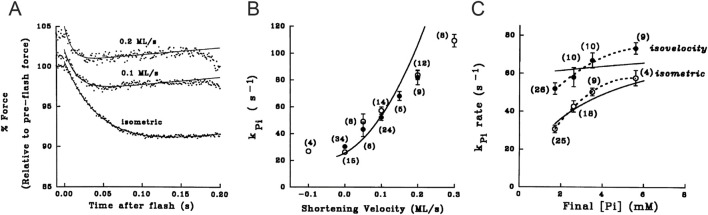
Pi transients in isometric contraction and during isovelocity shortening. **(A)** Force responses to Pi jump (points) imposed in isometric contraction and during shortening at the velocities (ML/s means fiber length/s) indicated on the traces. Records were interpolated by the sum of an exponential and a straight line (continuous line). **(B)** Relation of *k*_Pi_ versus shortening velocity. **(C)** Relation between *k*_Pi_ and final Pi concentration in isometric contraction (open circles) and during shortening at 0.1 fiber length/s (filled circles). Dashed lines interpolated to data. Continuous lines in **(B–C)** are the predictions of the model reported in Scheme 1 (reproduced from Figures 3, 4, 8 in Homsher et al. (1997) with permission).

To test the strain-dependence of the rate constants of step 3, the analysis of the Pi transient was extended to steady shortening at different velocities, thus reducing the load on the attached myosin motors in this way (Figure 8; Homsher et al., 1997). It was shown that *A*_Pi_ reduces with shortening velocity (Figure 8A), while *k*_Pi_ increases in proportion with the shortening velocity (Figure 8B) and, at a relatively slow shortening velocity, shows the same hyperbolic dependence on [Pi] as in the isometric contraction (Figure 8C). The increase in *k*_Pi_ and the reduction in *A*_Pi_ with increasing shortening velocity could be explained by introducing a strain-dependence of the rate constants of step 3 in the Reaction Scheme 1, as proposed by Dantzig et al. (1992). This model, however, was not able to fit the dependence of *k*_Pi_ on [Pi] during shortening, even at the low velocity used in Figure 8C (filled circles). As demonstrated with the model simulation reported in the final section, the explanation for the failure, which is common to all models with Pi release in series with the working stroke (Homsher et al., 1997; Ranatunga, 1999; Smith, 2014; Stehle and Tesi, 2017; Offer and Ranatunga, 2020), can be found in the assumption that Pi leaves the active site from a unique motor conformation either before or after the working stroke and, thus, with a unique value of its rate constant. Instead, a model where the working stroke and the Pi release are orthogonal processes (Caremani et al., 2013; Caremani et al., 2015) can fit the data in Figure 8C as it allows the Pi release rate to increase with the change in motor conformation during its progression through the working stroke.

## Matching the mechanics and energetics of shortening muscle requires a myosin motor to interact with two actin sites during one ATP hydrolysis cycle

**FIGURE 9:**
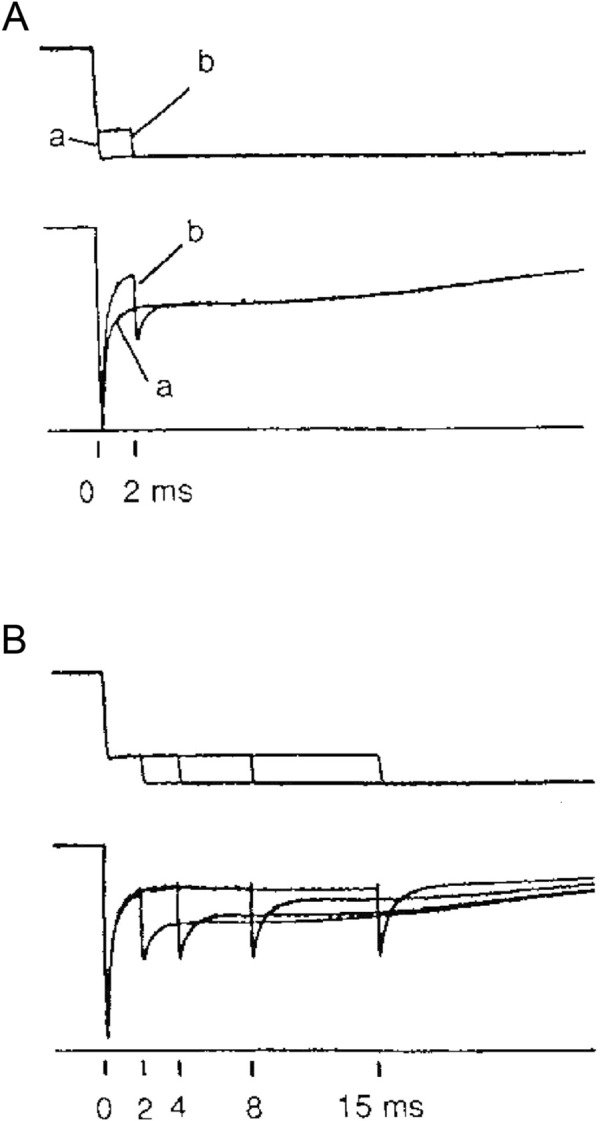
Force transient elicited by a step release imposed at different times following a conditioning step. **(A)** Superimposed records of force responses (lower traces) to (a) a single step release of 7 nm per half-sarcomere (upper traces) and to (b) a step of 2 nm delivered 2 ms after a conditioning step of 5 nm. **(B)** Superimposed records of force responses to test steps of 2 nm delivered 2, 4, 8, and 15 ms after the conditioning step of 5 nm. Single muscle fiber from *Rana esculenta* tetanically stimulated at 2.1 μm sarcomere length and 4 °C. Half-sarcomere length changes measured in a selected fiber segment measuring ∼1 mm in length. From Figure 1 in Lombardi et al. (1992).

The first evidence of a loose coupling between the mechanical and biochemical cycle of actin–myosin interactions during shortening was the finding that the working stroke on the attached myosin motors could be reprimed much faster than expected from the rate of the ATP hydrolysis (Lombardi et al., 1992). With double-step releases, each <5 nm per half sarcomere, superimposed on the isometric contraction (Figure 9), it was demonstrated that, if delivered within 1–2 ms following the first release, the second step release elicits a quick recovery that corresponds to that elicited by the sum of the two releases. Meanwhile, the quick recovery from a second step release imposed progressively later, increases and recovers the value expected from a single-step release with a time constant of ∼6 ms (at 4 °C), which is at least four times faster than expected from the kinetics of the attachment step, underpinning the rate of isometric force development from 0 (time constant ∼26 ms; Figure 3A, B in Linari et al. (2015)).

Structural evidence of the fast repriming of the working stroke was obtained shortly afterward using X-ray diffraction (Irving et al., 1992), exploiting changes in the intensity of the third-order myosin-based meridional reflection to track the changes in the orientation of the lever arm of the myosin motor. In isometric contraction, the motor lever is oriented nearly perpendicular to the filament axis. During the quick force recovery after the step release, it tilts toward the center of the sarcomere, marking the synchronous execution of the working stroke, and then, within the next 15 ms, it recovers the isometric conformation in parallel with the recovery of the ability to generate a second working stroke, which is much faster than expected from the rate of the isometric force development.

The problem that the rate of motor attachment responsible for the transition from 0 to *T*_0_ in isometric conditions is too low to account for the rate of the working stroke regeneration during shortening is further exacerbated by the finding that the kinetics of the attachment step underpinning the development of isometric force is too low to account for the maximum power developed during isotonic contractions against loads of approximately 1/3 *T*_0_ (Pertici et al., 2023). On the other hand, the increase in the rate constant for the motor attachment required to fit the maximum power would imply an increase in the rate of force development to values larger than those observed. A way to keep the rate of isometric force development as low as required by that observed, preserving the power of the shortening contraction, is to assume a specific geometrical hindrance in the isometric condition consequent to the mismatch between actin and myosin periodicities (Marcucci et al., 2021). In this case, however, the fit of the observed power produced during shortening implies that the ATP hydrolysis rate (or the rate of energy liberation) increases by 8–9 times with respect to the isometric rate. This is a major drawback of the model of Marcucci et al. (2021) because the observed rate of energy liberation during an isotonic contraction developing maximum power is much lower, ranging from 2 (mammalian muscle) to 4 (frog muscle) times larger the isometric energy rate (Pertici et al., 2023, and references therein).

As shown by the model of Marcucci et al. (2021), any model that attempts to simultaneously simulate the limited rate of isometric force development and the power during isotonic shortening by increasing the apparent rate of attachment during shortening under the assumption that more motors become available with shortening (Chen and Brenner, 1993) finds its limit in an exaggerated increase in the rate of energy consumption for the maximum power relative to the isometric rate. This same limitation also applies to the Huxley and Tideswell (1997) model, in which the first attached motor of a myosin dimer during shortening facilitates the attachment of the partner motor. Eventually, the hypothesis that the reduced rate of isometric force development can be explained by mechanosensing-based thick filament activation (Linari et al., 2015), masking the effect of a rate of attachment that is high enough to account for the power developed during isotonic contraction, is contradicted on the energetic basis. In fact, in this case, the underlying ATP hydrolysis rate during steady isometric contraction would be fivefold higher than that observed (Marcucci and Reggiani, 2016).

**FIGURE 10:**
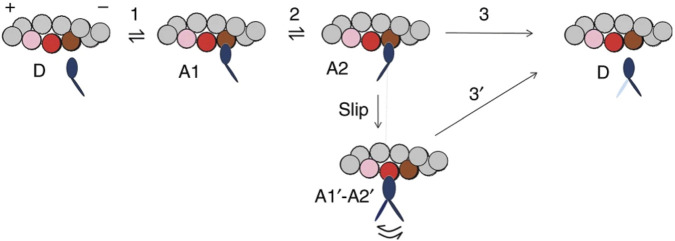
Simplified model with myosin slipping (Reaction Scheme 3). Kinetic scheme with three states of the myosin motor (blue): D, detached state; A1 and A2, low- and high-force states, respectively, attached to an actin monomer (brown). During shortening, the motor attached in the A2 state can slip to the next actin monomer farther from the center of the sarcomere (red) within the same ATPase cycle. The probability of a second slipping to the pink monomer is limited to 1/10 of that of the first slipping. From Figure 4 of Pertici et al. (2018).

The Reaction Scheme 3 (Figure 10) (Pertici et al., 2018) has the characteristics of a model that reconciles both the rapid regeneration of the working stroke determined in the transient regime (Lombardi et al., 1992) and the maximum power determined in the steady-state regime with the constraints that (*i*) the rate of isometric force development is four times slower than the rate of the working stroke regeneration following a step release and (*ii*) the rate of energy liberation in the isotonic contraction at the maximum power is no more than four times larger than that in the isometric contraction.

**FIGURE 11:**
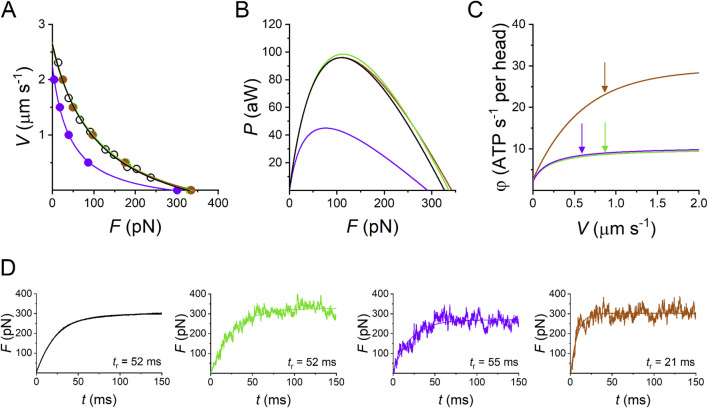
Model simulation of the performance of the half-thick filament of the frog skeletal muscle at 4.6 °C. **(A)** Force–velocity (*F–V*) relations. The force per half-thick filament (*F*, pN) is calculated from the force per cross-sectional area (*T*, with *T*_0_ = 165 kN m^-2^) taking a density of filament of 5.87 10^14^ m^-2^ (Piazzesi et al., 2018). Black circles and lines, experimental *F–V* relation; green circles and line, simulation with Reaction Scheme 3a, which is the integral version of Reaction Scheme 3 in Figure 10; violet circles and line, simulation with the suppression of slip transition (Reaction Scheme 3b); brown circles and line, simulation with the suppression of slip transition and *ad hoc* increase in the rate constants of attachment/detachment (Reaction Scheme 3c). **(B)** Power–force (*P–F*) relations calculated from data in A (same color code as in A). **(C)** Dependence of the rate of ATP hydrolysis per motor (φ) on *V*, according to the simulation with the model identified by the same color code as in **(A)**. Arrows indicate the values of φ at *V* for *P*_max_ (φ_*P*max_). In Reaction Scheme 3c, without slipping as in Reaction Scheme 3b, the increase in the attachment/detachment rate constants able to predict the experimental *P*_max_ implies an increase in φ_*P*max_ to ∼10 times the isometric value (the ordinate intercept). **(D)** Redevelopment of isometric force following a 5% rapid shortening able to decrease the isometric force *F*_0_ to 0. The rise time (from 10% to 90% of full change) is indicated next to the record. Black, observed (from Figure 3A green in Linari et al., 2015); simulated rises are identified by the color code as in **(A)** green (Reaction Scheme 3a), violet (Reaction Scheme 3b), and brown (Reaction Scheme 3c). Modified from Pertici et al. (2023).

The demonstration, summarized in Figure 11 and Table 1, is given for the performance of the frog skeletal muscle at 4 °C–5 °C (Pertici et al., 2023), but, as shown in the same paper, it is equally valid for the fast mammalian skeletal muscle.

**TABLE 1:** Simulations of the relevant mechanical and energetic parameters of the half-thick filament with Reaction Scheme 3a (integral version of the model shown in Figure 10), Reaction Scheme 3b (slipping suppressed), and Reaction Scheme 3c (slipping surrogated by *ad hoc* increase of the attachment–detachment kinetics). *F*_0_, steady isometric force per half-thick filament; φ_0_, steady isometric ATPase rate per myosin motor; *V*_0_, unloaded shortening velocity; *P*_max_, maximum power; φ_*P*max_, ATPase rate at *P*_max_; *t*_r_, rise time of isometric force development. Reaction Scheme 3a implies the possibility of the attached motor in state A2 to slip to the next Z-ward actin site. Reaction Scheme 3b retains the same kinetic scheme as Reaction Scheme 3a but excludes the possibility of slipping. In Reaction Scheme 3c, the attachment and detachment rate constants are increased to fit the observed *F–V* relation and *P–F* relation as shown in Figure 10. Data are presented as the mean ± SEM from at least 12 simulations. Table from Pertici et al. (2023).

*N* = 294 Available motors per htf	*F*_0_ (pN)	φ_0_ (s^-1^)	*V*_0_ (µm s^-1^)	*P*_max_ (aW)	φ_*P*max_ (s^-1^)	*t*_r_ (ms)
Reaction scheme 3a	336 ± 12	2.30	2.60 ± 0.07	98.5	8.63	52 ± 9
Reaction scheme 3b	291 ± 23	2.10	2.25 ± 0.03	45.0	8.45	55 ± 11
Reaction scheme 3c	342 ± 21	2.40	2.67 ± 0.11	96.0	23.1	21 ± 3

The three-state mechano-kinetic model of the actin–myosin interaction in Figure 10 has already been described in detail (Pertici et al., 2018; Pertici et al., 2020). The way in which the most relevant energetic features of the model are constrained by literature data is summarized here. In isometric contraction, the rate-limiting step in the cycle is detachment from the high-force generating state A2: the rate of ATP splitting per myosin motor (φ) is the minimum under the isometric conditions (φ_0_). During steady shortening, φ increases as the rate of motor detachment increases due to the accelerated execution of the working stroke. φ for the maximum power (φ_*P*max_) is higher than φ_0_ by a factor of four (Barclay et al., 2010). Under this condition, the curvature of the *F*–*V* relation and the resulting maximum power can be reproduced only by assuming the integral version of Reaction Scheme 3 (3a), in which, during shortening, the attached myosin motors can rapidly regenerate the working stroke during the same ATPase cycle by slipping to the next actin monomer farther from the center of the sarcomere (Lombardi et al., 1992; Piazzesi and Lombardi, 1995) (step “slip” in Figure 10) and undergoing A1′–A2′ state equilibration according to the strain dependency of step-2 kinetics (Huxley and Simmons, 1971). Detachment from either A1′ or A2’ (step 3′) implies ATP hydrolysis.

All the relevant emergent properties of the array of motors in the half-thick filament (htf) of fast skeletal muscle are reproduced by Reaction Scheme 3a. In Figure 11, the F–V relation (A), where *F* is the force per htf, is shown, and the *P–F* relations (B), simulated with Reaction Scheme 3a (green lines), are superimposed on the experimental data (black lines). The corresponding simulated φ–*V* relation, where φ is calculated by the flux through step 1, is shown in Figure 11C (green line). The relevant parameters are shown in the first line of Table 1. Notably, the observed *P*_max_ is simulated with φ (φ_*P*max_), which is four times larger than the value for the isometric force *F*_0_ (φ_0_).

The limits of the conventional mechano-chemical cycle in which the ATP hydrolysis is completed within a single actin–myosin interaction are summarized in the simulations of Reaction Scheme 3b and c, where the slipping transition is removed. If no other adjustments are introduced (Reaction Scheme 3b), *F*_0_ and φ_0_ are not substantially affected, while *P*_max_ is reduced to less than ½ (violet line in Figure 11B) without a marked change in φ_*P*max_ (violet arrows in Figure 11C; Table 1). With Reaction Scheme 3c, the mechanical performance of fast skeletal muscle (brown lines in Figures 11A, B) is recovered in the absence of slipping with *ad hoc* increases in the relevant rate constants for attachment and detachment (for the details of the kinetic assumptions, see Pertici et al., 2023). The consequences are that while φ_0_ can be maintained in substantial agreement with the experimental value, φ_*P*max_ (brown arrow in Figure 11C; Table 1) increases by ∼10-fold with respect to φ_0_.

The mechanical parameter that also in isometric conditions is sensitive to the kinetic differences between Reaction Scheme 3a and c is the rate at which force reaches the steady-state isometric value *F*_0_. This is expected from the consideration that the time course of force increase depends on the rate constants governing attachment (step 1) and force generation (step 2) and, specifically, in this model, on step 1, which is the rate limiting step in the process. To avoid the influence of the time required for thick filament activation on initial force development (Linari et al., 2015), the selected reference parameter is the force redevelopment following a 5% rapid shortening, which can reduce and maintain the isometric force at 0 (Figure 11D, black trace). The rise time *t*_r_ (the time from 10% to 90% of *F*_0_), estimated from the exponential fit to the recorded trace, is 52 ms, and it is perfectly reproduced by the simulation with Reaction Scheme 3a (Figure 11D, green trace, and first row in Table 1). In contrast, *t*_r_ is reduced to less than ½ in the simulation with Reaction Scheme 3c (Figure 11D, brown trace, and third row in Table 1), demonstrating that Reaction Scheme 3c fails to predict the observed low rate of the attachment-force development. Notably, *t*_r_ simulated by Reaction Scheme 3b (Figure 11D, violet trace, and second row in Table 1) fits the observed value. This is because Reaction Scheme 3b shares the same kinetic scheme as Reaction Scheme 3a, apart from the slipping transition, which is effective only for the performance of the shortening muscle.

## Chemically and structurally explicit model where Pi release is orthogonal to the working stroke transition

**FIGURE 12:**
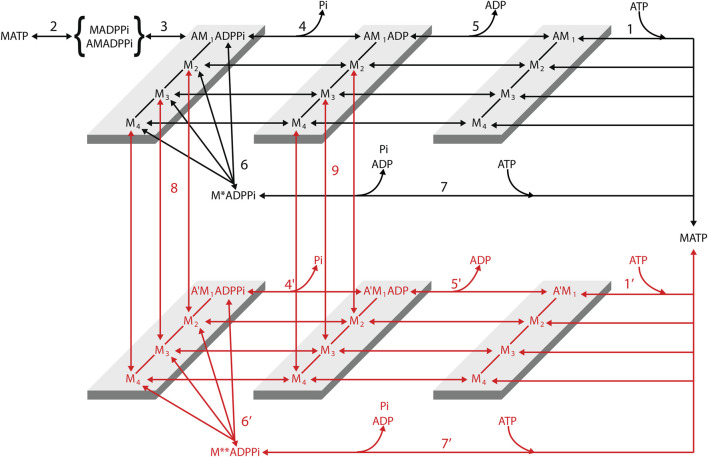
Structurally and chemically explicit cycle of the myosin motor integrating the properties of the three preceding schemes (Reaction Scheme 4). Transitions shown in black indicate the cycle undergone by myosin motors (M) that interact with only one actin monomer (A). Transitions shown in red indicate the cycle undergone by myosin motors slipping to the next actin monomer 5.5 nm away from the center of the sarcomere (A′). For simplicity, this legend is mostly limited to black transitions. M_1_–M_4_ represent the structural states defining the progression of the working stroke in a given biochemical state of the attached myosin motor. Binding of an ATP molecule induces dissociation of the myosin motor from actin (step 1, 1′), which is followed by the recovery of the conformation of the motor at the beginning of the working stroke and the hydrolysis step (step 2). The MADPPi–AMADPPi state represents both the detached motor just after the hydrolysis step and recovery stroke and the weakly actin-bound motor with the hydrolysis products still bound. Strong binding of M to an actin monomer A (step 3) implies the formation of the first of four different force-generating states, AM_1_ADPPi, which, without significant delay, undergoes the structural transition, leading to the strained conformation responsible for the isometric force. The working stroke, in any of the biochemical states, AMADPPi, AMADP, and AM, implies three subsequent force-generating steps (M_1_→ M_2_, M_2_→ M_3_, and M_3_→ M_4_) controlled by strain-dependent rate constants (Huxley and Simmons, 1971). Both biochemical events in the attached motor, release of Pi (step 4, 4′) and release of ADP (step 5, 5′), can occur in any of the four structural states. Unconventional pathways are represented by the possibility of the force-generating AMADPPi state to detach (step 6, 6′) and rapidly release the hydrolysis products and rebind ATP (step 7, 7′) and the possibility that during shortening, the motor attached to the first actin monomer (A) slips to the next Z-ward actin monomer (A′, steps 8 and 9). This possibility is specifically relevant to the AMADP state (step 9; see the rate functions listed in Supplementary Table 1). Reproduced from Figure 1 in Caremani et al. (2015) with permission.

Reaction Scheme 4 (Figure 12) (Caremani et al., 2013; Caremani et al., 2015) integrates the properties of Reaction Schemes 1–3, described in the previous sections, into a chemically and structurally explicit model that retains the predictive properties of the previous schemes and adds the possibility to both simulate the velocity transients elicited by step perturbations in force and their modulation by increase in [Pi] and identify the biochemical states that are involved in unconventional pathways such as early detachment from a force-generating state or, during shortening, slippage to the next actin monomer away from the sarcomere center.

The following is a summary of the Reaction Scheme 4. The two-step reaction responsible for force generation and release of Pi in Reaction Scheme 1 (steps 3 and 4, respectively), predicting the hyperbolic dependence of *k*_Pi_ (but also of *k*_TR_) on [Pi] (Figure 7C), is reproduced in Reaction Scheme 4 (Figure 12) by steps 3 and 4 (and 4′). The early detachment from the force-generating AM'ADPPi state and the subsequent rapid release of the hydrolysis products in Reaction Scheme 2 (steps 6 and 7, respectively), which are required to explain the reduced effect of the increase of [Pi] on the ATPase rate (Figure 7B), are reproduced in Reaction Scheme 4 by steps 6 (and 6′) and 7 (and 7′), respectively. The slipping of an actin-attached motor during shortening to the next actin monomer farther from the sarcomere centre within the same ATP hydrolysis cycle (step “slip” in Reaction Scheme 3a, Figure 11), required for a rapid regeneration of the working stroke (Figure 9) and for the observed maximum power (Figure 11B) with a limited increase in the rate of energy liberation (Figure 11C) during steady shortening, is reproduced in Reaction Scheme 4 by steps 8 (slipping from the AMADPPi states) and 9 (slipping from the AMADP states).

**FIGURE 13:**
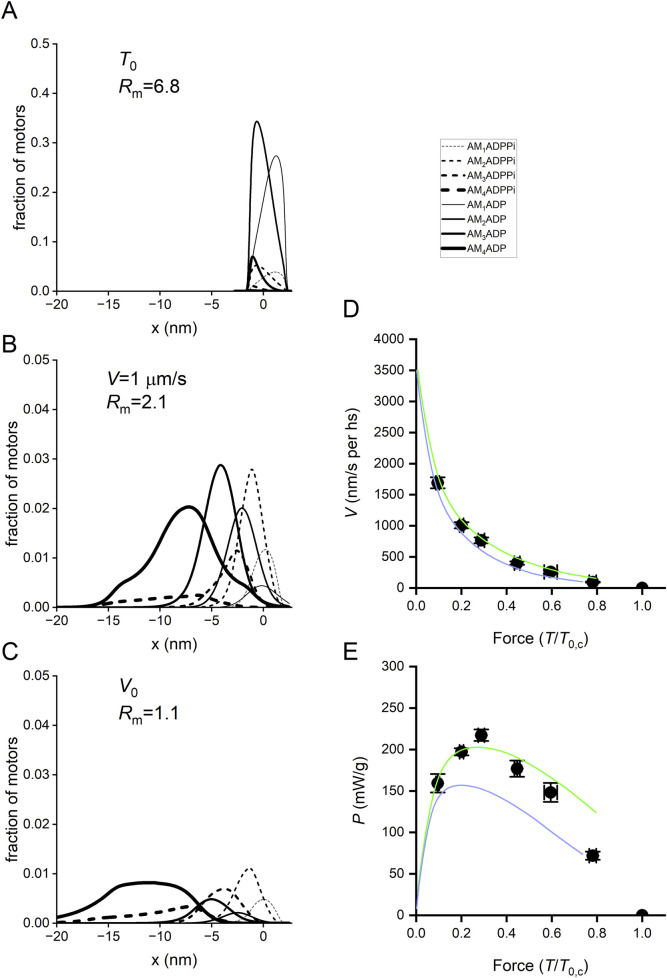
x-distribution of the attached states in isometric contraction and during shortening and simulation of the force–velocity and power–force relations with Reaction Scheme 4. **(A-C)** x-distribution of the attached states in isometric contraction and during shortening. Dashed lines, AMADPPi states; continuous lines, AMADP states. Progression of states through the working stroke identified by the progression from the thinnest to the thickest line, as indicated in the inset. **(A)** Distribution at *T*_0_. *R*_m_, the ratio between AMADP and AMADPPi motors, is ∼7. **(B)**. Distribution during isotonic shortening at *V* = 1,000 nm/s per hs, *R*_m_ is ∼2. **(C)**. Distribution at *V*_0_, *R*_m_ is ∼1. **(D)** *T–V* relations. Circles, experimental data; lines, simulated relations with Reaction Scheme 4 with (green) and without (violet) steps 8 and 9. **(E)** *P–T* relations calculated from *T–V* relations in **(D)**. Circles and lines, same codes as in **(D)**.

The biochemically different force-generating states of Reaction Scheme 1 that are significantly populated in physiological conditions (AMADPPi and AMADP) in Reaction Scheme 4 exist in several conformations depending on the progression through the working stroke. Strong binding of M to an actin monomer A (step 3), caused by the closure of the actin-binding cleft (Geeves and Holmes, 2005), implies the formation of the first, AM_1_ADPP_i_, of several states generating progressively higher force. The kinetic, mechanical, and energetic features constraining the simulation of the working stroke have already been described in detail for the intact frog muscle fiber (Figure 4). In that case, the stiffness of the motor was 3 pN/nm so that the size of the force-generating transition, corresponding to the progressive swinging of the lever arm, was set to 2.75 nm (Piazzesi et al., 2014), and the number of transitions for an 11-nm working stroke was four (Figure 4D). In the case of the mammalian-skinned fiber, the smaller stiffness of the motor (≤1.7 pN/nm; Linari et al., 2007) allowed the assumption of a larger size of the transition (3.1 nm) and only three force-generating transitions, namely, M_1_→ M_2_, M_2_→ M_3_, and M_3_→ M_4_. According to the principle of the nearest-neighbor interaction, attachment to the actin monomer (with approximately 5.5-nm axial separation between monomers along a strand of the actin helix) occurs for a range of *x* from −2.75 nm to 2.75 nm, where *x* is the relative axial position between the motor and A, and is 0 for the position of the center of distribution of attachments of the motors in the M_1_ state (Figure 13A). AM_1_ADPPi undergoes the first transitions leading to the strained conformation responsible for the isometric force (Figure 13A).

Both the release of P_i_ (step 4) and the release of ADP (step 5) can occur from any of the four attached states, that is P_i_ release and and ADP release are orthogonal to the working stroke state transitions. The most important consequences for the aims of this report are as follows: first, the working stroke kinetics is independent of the concentration of Pi, as shown from the data in Figure 6D; second, the kinetics of Pi release can be made to depend on the conformation of the motor, increasing with the progression of the motor through the working stroke from M_1_ to M_4_. Notably, this kinetic feature is excluded in all the reaction schemes in which Pi-release coincides (Pate and Cooke, 1989b) or is in series (Dantzig et al., 1992; Homsher et al., 1997; Ranatunga, 1999; Smith, 2014; Stehle and Tesi, 2017; Offer and Ranatunga, 2020) with the force-generating transition. The rate functions of the reaction scheme are given in Supplementary Table 1. Further details on the calculation procedure can be found in Caremani et al. (2013) and Caremani et al. (2015).

Reaction Scheme 4 (Figure 12) retains all the properties of the three preceding schemes, and most of the mechanical and energetic outputs, observed for mammalian skinned fibers both without and with added P_i_, are reported by Caremani et al. (2013). Therefore, its predictive power is demonstrated in detail here, mainly for the transient mechanical properties not simulated in the previous sections. In addition, with Reaction Scheme 4, as already demonstrated with the simplified Reaction Scheme 3 (Figures 11A, B), suppressing the possibility for an attached motor to slip to the next actin monomer farther from sarcomere centre during shortening increases the curvature of the *T–V* relation (Figure 13D, violet line) and reduces the maximum power (E, violet line) with respect to the experimental data (circles).

**FIGURE 14:**
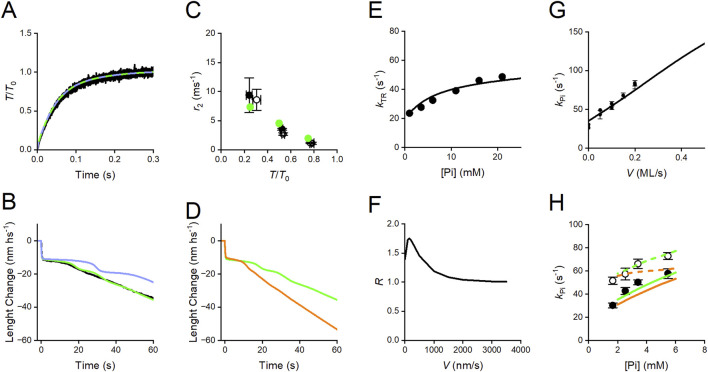
Simulation of transient responses and the effect of Pi in skinned fibers from rabbit psoas with Reaction Scheme 4. **(A)** Superimposed time course of transition from 0 to *T*_0_ in the isometric contraction. Black trace, experiment; green trace, simulation with the complete scheme; violet trace, simulation after suppression of slipping possibility (steps 8 and 9). **(B)** Superimposed velocity transients elicited by a force drop to 0.5 *T*_0_. Black trace, experiment; green and violet traces, simulation with and without slipping, respectively. **(C)** Dependence on *T*/*T*_0_ of the rate constant of the working stroke (*r*_2_) elicited by a stepwise decrease in force. Black circles, experimental data from Figure 5D without (filled) and with 10 mM added Pi (open); green circles, simulation without (filled) and with 10 mM added P_i_ (open, masked by the filled circles). **(D)** Simulation of the effect of 10 mM Pi on the velocity transient elicited by a force drop to 0.5 *T*_0_ without (green) and with (orange) 10 mM added Pi. **(E)** Dependence on [Pi] of *k*_TR_, the rate of the transition from 0 to *T*_0_ in the isometric contraction. Circles, experimental *k*_TR_–Pi relation from Figure 7C; line, simulated relation. **(F)** Dependence on the shortening velocity of the ratio of the rate of ATP hydrolysis per motor with 10 mM added Pi over that without (*R*). **(G)** Relation between the rate of the Pi transient, *k*_Pi_, and the shortening velocity. Dots, experimental data from Figure 8B; line, model simulation. **(H)** Relations between *k*_Pi_ and final [Pi] in isometric contraction (filled circles) and during shortening at 0.1 fiber length/s (open circles) from Figure 8C. Continuous and dashed lines, simulations fitted to the data in isometric condition and during shortening, respectively. Green, simulations with Reaction Scheme 4; orange, simulation with the same reaction scheme as green but with the assumption of the same value (100 s^-1^) for the rate constant of the Pi release (*k*_4_) for the four conformations.

Again, as shown in the Reaction Scheme 4, the rate of transition from 0 to the steady-state isometric force is not affected by the suppression of the slipping possibility (Figure 14A; black trace, experimental record; green and violet lines, force rise simulated with Reaction Scheme 4 with and without steps 8–9, respectively).

Reaction Scheme 4 is unique in allowing simulation of the force and velocity transients elicited in response to a step in length or load that synchronizes the execution of the working stroke and its dependence on mechanical and biochemical conditions. As already discussed, the presence of a significant compliance in the myofilaments, functionally in series with the motor array in each half-sarcomere, complicates the use of the rate of quick force recovery as a constraint for modeling the working stroke kinetics (Piazzesi et al., 2014). Instead, phase-2 shortening elicited by a stepwise decrease in force (Figures 3B, 5D) directly provides information on the load dependence of the rate and the size of the working stroke, and the following phase-3 pause provides information on the kinetics of the events leading to working stroke regeneration. Reaction Scheme 4 can satisfactorily fit the isotonic velocity transient (Figure 14B: black, experiment; green, model), providing a direct demonstration of the role of slipping in the rapid regeneration of the working stroke, which is the condition for the maximization of the power during the more physiological steady shortening. Following the removal of steps 8–9, the phase-3 duration increases (Figure 14B, violet), delaying the transition to the steady shortening velocity of phase 4, with consequences on the steady-state (physiological) performance, such as the increase in the curvature of the force–velocity relation and the reduction in the power output (violet lines in Figures 13D, E). As already discussed for the simplified Reaction Scheme 3, this drawback, intrinsic to any conventional chemo-mechanical cycle, cannot be remedied by increasing the rate constant for the attachment step, because of the limit imposed to the kinetics of this step by the observed rate of isometric force development (Figures 11D, 14A).

A consequence of the idea that the release of Pi is orthogonal to the working stroke transitions is that only the kinetics of the working stroke is strain-dependent. Instead, Pi-release kinetics is sensitive to the structural state of the attached motor (conformation-dependent) and, thus, indirectly connected to the mechanical conditions by the speed of the progression through the different conformations (M_1_–M_4_). Under these conditions, the model satisfies the constraints that the speed of the working stroke (the rate of phase-2 rapid shortening, *r*_2_) elicited by a stepwise force decrease (*i*) increases with the size of the step (Figure 14C: filled symbols: black, experiment; green, model) and (*ii*) is not affected by the increase in [Pi] (Figure 14C; open symbols: black, experiment; green, model).

With smaller steps, i.e., at higher loads, the working stroke is slower (Figures 3B, 5D), which increases the probability that the other reactions taking over in the phase-3 pause (early detachment with the hydrolysis products in the catalytic site (step 6) and slipping to the next actin monomer (steps 8 and 9)) truncate the working stroke and lead to its regeneration. An increase in [Pi] alone also truncates the working stroke and accelerates phase-3 pause for the same relative decrease in force (Figures 6A–C, E). Notably, this effect is accompanied by an increase in both *k*_Pi_ and *k*_TR_ (Figure 7C), revealing that the involved kinetic steps are not restricted to the unconventional early detachment but also include the conventional cycle.

Reaction Scheme 4 can discriminate two different mechanisms leading to the accelerated working stroke regeneration during shortening: (*i*) at low Pi, the operating mechanism is based on the possibility that, for critical negative values of x, the force-generating motors, mainly those in the AMADP state, slip to the next Z-ward actin monomer (step 9), reducing phase-3 pause (Figure 14B, green) and, in this way, increasing the sliding distance per one ATPase cycle (see Figure 7A in Caremani et al., 2013), without any effect on *k*_TR_ (Figure 14A); (*ii*) the operating mechanism induced by the increase in Pi (Figure 14D: green, no added Pi; orange, 10 mM Pi) is based on the acceleration of the whole attachment-force generation and detachment cycle, as revealed by a corresponding increase in *k*_TR_ (Figure 14E; circles: experiment, line: model simulation), as a consequence of the increase in the second-order rate constant of the reversal of Pi release. Under these conditions, the model predicts that, in isometric contraction and at high loads, an increase in [Pi] increases the rate of ATP hydrolysis per motor (Figure 14F), which is the tension cost.

**FIGURE 15:**
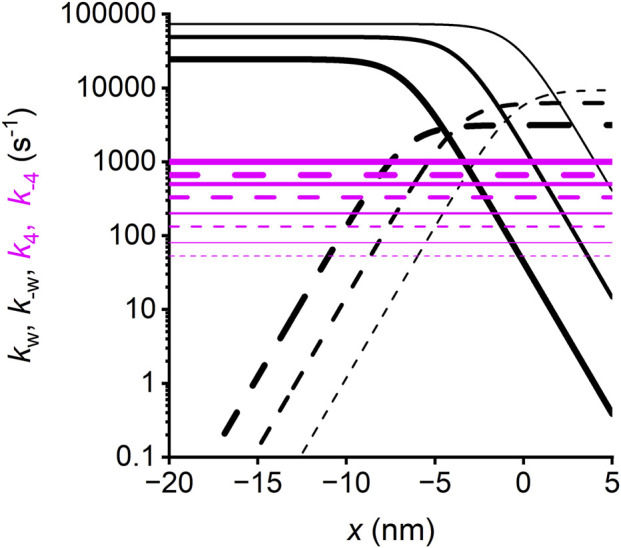
Strain-dependence of the rate constants of the working stroke transitions and Pi release. Forward transitions, continuous lines; backward transitions, dashed lines. Black, working stroke transitions; line thickness increases with the progression of the transition from the first (M_1_–M_2,_ the thinnest line) to the third (M_3_–M_4_, the thickest line). Magenta, Pi release step; line thickness increases with the progression of motor conformation from M_1_ (the thinnest line) to M_4_ (the thickest line). For simplicity, the transitions considered are those concerning the myosin motor while attached to the first actin monomer A (and the slip to the next actin monomer A′, 5.5 nm away from the sarcomere center can be deduced by shifting the rate functions for A leftward by 5.5 nm).

Conventional models in which attachment-force generation is strain-dependent can predict the finding from Pi jump experiments that the rate of the Pi transient is larger during isovelocity shortening than under isometric conditions (Figure 8A) and increases with an increase in shortening velocity (Figure 8B). However, those models cannot predict that during isovelocity shortening, the rate of Pi transient increases with an increase in final [Pi], as in the isometric contraction (Figure 8C). This is because in all those models, the Pi release is a step in series with the working stroke and thus can be defined by only one rate constant. In Reaction Scheme 4, Pi release is orthogonal to the working stroke, and its kinetics depends on the effects that the progression of the working stroke exerts on the catalytic site. Assuming that the rate of Pi release (*k*_4_) increases with the transition of motor conformation from M_1_ to M_4_ (Figure 15, see also Supplementary Material) is the sole condition that allows the rate of Pi transient to depend on either the velocity in isovelocity contractions (Figure 14G) or the final [Pi] both in isometric contraction (Figure 14H, green continuous line) and during shortening (green dashed line), as observed (circles, filled during isometric contraction, and open during shortening). If *k*_4_ is given a convenient constant value (100 s^-1^), the simulation still predicts *k*_Pi_ to depend on the final [Pi] in isometric contraction (orange continuous line) but not during shortening (orange dashed line).

## Contribution of Reaction Scheme 4 in clarifying unsolved questions of the coupling between the myosin working stroke and Pi release

### Kinetic aspects

First, the structurally and biochemically explicit Reaction Scheme 4 allows clarifying the longstanding issue of the relative timing between the myosin working stroke and Pi release in fast skeletal muscle. According to this scheme, the release of Pi can occur at any stage of the working stroke with a conformation-dependent kinetics, implying that the rate of Pi release increases with the transition of motors from the M_1_ to M_4_ states (Figure 15, magenta lines). Reaction Scheme 4, first published in 2013 (Caremani et al., 2013), resolves all contradictions inherent to conventional models in which Pi release occurs in series with the working stroke, either before (Smith, 2014; Offer and Ranatunga, 2020) or after the stroke (Kawai and Halvorson, 1991; Dantzig et al., 1992; Homsher et al., 1997). Both hypotheses have the limitation that they do not explain (*i*) why the increase in [Pi] decreases the number of attached/force generating motors much more than the ATPase rate and (*ii*) why Pi transient kinetics depends on [Pi] both in the isometric contraction and during shortening. Moreover, the hypothesis that Pi is released before the working stroke has the further contradiction that the working stroke kinetics depends on the concentration of Pi (see Supplementary Figure 5 in Moretto et al., 2022).

The powerful tool offered by a structurally explicit model is the possibility to test the consequences on the muscle performance of the idea that the Pi release rate is conformation-dependent. This exquisitely emerges from the analysis of the fractional occupancies of the two biochemically distinct attached states in relation to the load of the contraction (Figure 13): at *T*_0_, the distribution of the attached motors is around x = 0 and is biased toward the early structural states (mostly M_1_ and M_2_, Figure 13A; the thinnest and second thinnest lines) by the strain-dependent kinetics of the working stroke transitions (Figure 15, black lines; see Caremani et al., 2015, while the Pi release kinetics (magenta lines), although relatively slow, favors the AMADP state). The ratio between the AMADP and AMADPPi states (*R*_m_) is 6.8. During isotonic shortening at *V* = 1 μm/s per hs (close to that for the maximum power in the skinned rabbit fiber) (Figure 13B), the motor distribution spreads toward negative values of x, favoring the transition toward structural states ahead in the working stroke (M_3_ and M_4_). Because of the conformation-dependent increase in the rate of Pi release, the result is a progressive shift from the AMADPPi state to the AMADP state of the M_3_ and M_4_ motors. At the same time, the Pi release becomes the rate-limiting step for the short-lived M_1_ and M_2_ motors. The result is that the fractional occupancy of the AM_1_ADPPi state (the thinnest dashed line) and the AM_2_ADPPi state (the second thinnest dashed line) is higher than the corresponding AM_1_ADP (the thinnest continuous line) and AM_2_ADP (the second thinnest continuous line) states, respectively, while the proportion is inverted for the M_3_ and M_4_ motors (the second thickest and thickest lines, respectively). Altogether, *R*_m_ decreases to 2.1. *R*_m_ further reduces to 1.1 for the maximum shortening velocity (*V*_0_ = 3.5 μm/s per hs, Figure 13C). This is because *V*_0_ implies a further shift of the distribution toward negative x and, thus, even faster strain-dependent working stroke transitions. Consequently, Pi release becomes the rate-limiting step for all motor conformations, which are mostly in the AMADPPi state, except M_4_, for which the fractional occupancy of the AMADPPi state (the thickest dashed line) progressively decreases for more negative x with respect to that of the AMADP state (the thickest continuous line). This is because M_4_, representing the final working stroke conformation, becomes a progressively more long-lived state as it attains more negative x. At *V*_0_, the distribution of attached motors extends well beyond x = −10 nm, and an M_4_ motor requires more than 3 ms to reach values beyond −10 nm, a time three times longer than the time constant for the release of Pi.

The inhibitory effect of the increase in [Pi] on the number of force-generating motors is much higher than that on the ATPase rate and is explained in Reaction Scheme 4 with the presence of an unconventional short pathway for the completion of the ATPase cycle, consisting of the early detachment of the motors from the force-generating AMADPPi states (step 6 in Figure 12), followed by rapid and irreversible release of the hydrolysis products and binding of a new ATP (step 7). Under this condition, the flux through step 6 is intrinsically sensitive to any increase in [Pi] through the mass action exerted by the second-order rate constant of the reversal of step 4. This pathway reflects its effects on the Pi transient kinetics, weakening the interpretation of Pi transients with the two-step reaction expressed by Equation 1.

The role of the two-step reaction is further undermined by considering the refined study of the Pi transient kinetics, which was made possible by the use of rabbit psoas myofibrils (Tesi et al., 2000). Unlike skinned fibers, in which a stepwise increase in [Pi] can be imposed by photo-liberation of caged Pi, in myofibrils, stepwise changes in [Pi] in either direction can be achieved by a rapid exchange system made by a double-barreled pipette. It was found that both the rate of the decrease in force elicited by a stepwise increase in [Pi] (*k*_+Pi_) and its hyperbolic dependence on the final [Pi] were similar to those observed with Pi jumps in skinned fibers. Instead, the rate of the force rise elicited by a stepwise reduction in [Pi] (*k*_-Pi_) was 2–3 times lower than that of the force reduction elicited by a stepwise increase to the same final [Pi]. In addition, the relation expressing *k*_‐Pi_ dependence on the final [Pi] was shifted downward with respect to the *k*_+Pi_-Pi relation, becoming similar to the *k*_TR_–Pi relation characterizing the force redevelopment from 0 following a release superimposed on the isometric force. Two questions emerge from these results, both challenging the solidity of the interpretation of the Pi transient with a two-step reaction scheme. The first concerns the asymmetry between *k*_+Pi_ and *k*_-Pi_, which is unexpected on the basis of the two-step reaction; the second concerns the finding that *k*_-Pi_ exhibits the same kinetics as *k*_TR_ and, thus, overcomes the limits of the two-step reaction, encompassing the whole attachment–detachment cycle. An explanation for the finding that *k*_+Pi_ > *k*_-Pi_ (and k_TR_) was that, in analogy with the rapid phase of force relaxation following the decrease in activating Ca^2+^, the decrease in force in the isometric condition, whatever the reason is, it is accompanied by the development of sarcomere inhomogeneity and formation of weak sarcomeres that speed-up force decrease (Stehle, 2017). However, this argument was contradicted by evidence from Tesi et al. (2000), showing that the force transient following Ca^2+^ or ADP jumps in either direction did not show any direction-dependent kinetics. Moreover, while *k*_-Pi_ and k_TR_ were sensitive to the concentration of activating Ca^2+^, as expected for a reaction scheme implying *de novo* motor attachment (Brenner, 1988), *k*_+Pi_ was not. These results (*i*) exclude the possibility to explain the Pi transient following an increase in [Pi] simply with an increase in the flux through the reversal of the attachment step (step 3 in Figure 12)—a possibility that is also excluded by the evidence that an increase in [Pi] decreases the force and the number of attached motors much more than the ATP turnover (Webb et al., 1986; Bowater and Sleep, 1988; Cooke et al., 1988; Potma et al., 1995; Potma and Stienen, 1996; Caremani et al., 2008)—and (*ii*) suggest that the increase in [Pi] promotes the flux through a detachment path different from the reversal of attachment and committed for ATP hydrolysis, as the alternative cycle through step 6. If this unconventional pathway is irreversible, the increase in [Pi] by mass action would promote the increase in the flux through both the conventional (reversal of step 3) and the unconventional pathways (steps 6 and 7), leading to a faster Pi transient. Moreover, the finding that *k*_-Pi_ is smaller than *k*_+Pi_ and similar to *k*_TR_ could be explained according to Reaction Scheme 4 by considering that a reduction in [Pi] would promote only *de novo* attachment through the conventional pathway. According to the ^18^O exchange experiments, the Pi release step is reversible in skinned fibers but not in actomyosin S1 in solution (Bowater and Sleep, 1988). The same irreversibility could characterize the release of Pi from the unconstrained MADPPi state generated by the detachment of the force generating AMADPPi motors (Figure 12). In conclusion, for fast skeletal muscle, any alternative model (Stehle, 2017; Mansson, 2025) based on the assumptions that *k*_Pi+_, k_Pi-_, and *k*_TR_ are similar and the asymmetry in the Pi transient arises from an increase in sarcomere inhomogeneity with force decrease is not sustained by experimental evidence. Instead, the idea that the asymmetry of the Pi transient kinetics in fast skeletal muscle is because of an early detachment of force-generating motors with the hydrolysis products, introduced to explain the limited effect of the increase in [Pi] on the rate of ATP hydrolysis (Linari et al., 2010), is further supported by the behavior of these parameters in slow (soleus) skeletal muscle. It was found that at temperatures ≤15 °C, the increase in [Pi] has a similar inhibitory power of isometric force and ATPase rate (Potma et al., 1995; Kerrick and Xu, 2004). In this case, one expects that the probability of a flow through the unconventional cycle remains low independent of Pi. Accordingly, in the same preparation, it was found that there is no asymmetry in the Pi transient kinetics, and both *k*_+Pi_ and *k*_-Pi_ are similar to *k*_TR_ (Millar and Homsher, 1992; Stehle and Tesi, 2017).

### Structural aspects

Crystallographic, EM, and transient biochemical studies of the decade across the end of the last century (Rayment et al., 1993a; Zeng et al., 2004; Geeves and Holmes, 2005) provided the classical synthetic description of the relevant molecular aspects of actin attachment and force generation mentioned in the Introduction section (Figure 2). The transition of the interactions between myosin S1 and actin from weak to strong, with the closure of the cleft between the upper and lower 50 kDa domains, is the trigger for the change in a joining structure (switch 1) that induces both the release of Pi from the active site and the rotation of the converter-tilting of the lever arm (Figure 2). This scenario has been challenged by a more recent crystallographic study on non-muscle myosin VI integrated with site-specific mutations and kinetic measurements (consisting of rapid freezing of the crystal soaked in high [Pi] for different times) (Llinas et al., 2015). It is shown that Pi leaves the active site before the complete closure of the cleft (recorded by quenching of actin pyrene label; De La Cruz et al., 1999) because the early changes promoted by the formation of the actin–myosin interface trigger changes of another switch (switch 2) that opens the way for Pi to leave the active site from the backdoor. Thus, an intermediate state (Pi release state, PiR) precedes the cleft closure, switch 1 movement, and the execution of the working stroke. The new scenario includes the presence of possible interactions of Pi within the tunnel that leads to the backdoor, which would delay the appearance of Pi in solution with respect to the time it leaves the active site. Therefore, even if the working stroke is promoted by Pi leaving the active site, Pi may appear in solution after the end of the working stroke, especially at a low load, which explains the results of the FRET experiment (Muretta et al., 2015).

How do the mechanical and kinetic features of Reaction Scheme 4 integrate with this new structural scenario linking Pi release and the working stroke? The first question concerns the identification of the functional properties of the PiR state. A hypothesis promoted by the new crystallographic data (Llinas et al., 2015; Robert-Paganin et al., 2020; Moretto et al., 2022; Mansson, 2025) is that the PiR state represents the first attached state that triggers further cleft closure and the working stroke, while Pi progression through the tunnel still allows the reversal of Pi release. Taking into account the finding that the force and number of attached motors change in proportion during the increase in isometric force (Brunello et al., 2006; Caremani et al., 2008), it seems excluded that this state is a strongly attached, stiffness-generating state unless it coincides with the AM_1_ADPPi state in Figure 12, which is in rapid equilibrium with the higher force-generating states. On the other hand, if it is a weakly bound state, it coincides with the AMADPPi state, upstream with respect to step 3, which we cannot distinguish from the MADPPi state.

It must be noted that identifying the PiR state with the first attached state and, thus, with the AM_1_ADPPi state implies a contradiction with the results of sarcomere-level mechanical experiments on the effect of temperature on the force-generating transition of attached motors (Decostre et al., 2005; Linari et al., 2007). In those experiments, it was demonstrated that increasing the temperature potentiates the force developed by a motor during isometric contraction, at the expense of the size of the working stroke elicited by a stepwise decrease in force to zero superimposed on the steady isometric contraction. This indicates that a myosin motor *in situ* exploits the same working stroke mechanism for both the limited movement, underpinning the increase in strain and force in isometric conditions, and the maximum movement, underpinning the sliding under low load.

The second question concerns whether the conformation dependence of the Pi release rate can be structurally explained by the interactions of Pi within the tunnel after its release from the active site. This would be the case if the interaction kinetics and, thus, the time taken to appear in solution depended on the degree of progression of the working stroke.

In a first attempt to exploit the Llinas et al. crystallographic model for a kinetic “multistep Pi-release” model (Moretto et al., 2022), it is assumed that the working stroke may occur starting not only from the PiR state but also from any of the Pi states following the PiR state or from the AMADP state. In this respect, the model supports the idea proposed by Caremani et al. (2013) that the working stroke and Pi release are independent processes, with the simulated working stroke appearing unaffected by Pi. However, the simulation of the working stroke under unloaded conditions is not obtained by perturbing the equilibrium distribution of motors at the steady state of isometric contraction but by starting from a pre-working stroke state. Under this condition, the output of a “multistep Pi-release” model should consist of a mixture of working strokes equal to the number of states from which the working stroke occurs, and if they are separated by a time-consuming and Pi-sensitive process, such as the progression of Pi through the tunnel, the strokes cannot occur in parallel, and the resulting movement would be temporally dispersed and Pi-sensitive. Presently, the Llinas et al. (2015) crystallographic model is still untested for its functionally sound aspects in relation to the multifaceted mechanical and kinetic aspects of the *in situ* actin–myosin chemo-mechanical coupling recollected in this report, such as (*i*) the load dependence of the rate of the working stroke, (*ii*) the conformation dependence of the rate of Pi release accounting for the Pi transient kinetics, (*iii*) the much larger inhibitory effect of the increase of [Pi] on the number of attached motors than on the ATPase rate in contractions at high load, and (*iv*) the limited increase in the rate of energy consumption to achieve the observed power production during shortening.
