# Supplementary material for: Multiple pathways of the actin-myosin cycle in energy transduction and the release of orthophosphate in muscle
Source: Front Physiol. 2025 Nov 4;16:1664568. doi: 10.3389/fphys.2025.1664568 (PMC12623182; doi:10.3389/fphys.2025.1664568)
Supplement: Supplementary file 1 [file DataSheet1.doc]

Supplementary Material

# The multiple pathways of the actin-myosin cycle of energy transduction and the release of orthophosphate in muscle

Marco Caremani^1,2^, Irene Pertici^1,2^, Ilaria Morotti^1,2^, Pasquale Bianco^1,2^, Massimo Reconditi^1,3^, Gabriella Piazzesi^1^, Vincenzo Lombardi^1^, Marco Linari^1,2^

^1^PhysioLab, University of Florence, 50019 Sesto Fiorentino, Florence, Italy

^2^Department of Biology, University of Florence, 50019 Sesto Fiorentino, Florence, Italy

^3^Department of Experimental and Clinical Medicine, University of Florence, 50134 Florence, Italy

**Experimental set-up**

The set-up for sarcomere-level mechanics with nanometer-microsecond resolution has been first developed for intact fibres of frog muscle and then applied to skinned fibres from mammalian muscle . The fibre is mounted in a thermoregulated aluminium trough filled with physiological solution and is attached, via hooks, between the levers of a loudspeaker motor and a force transducer (Supplementary Figure 1A).

| 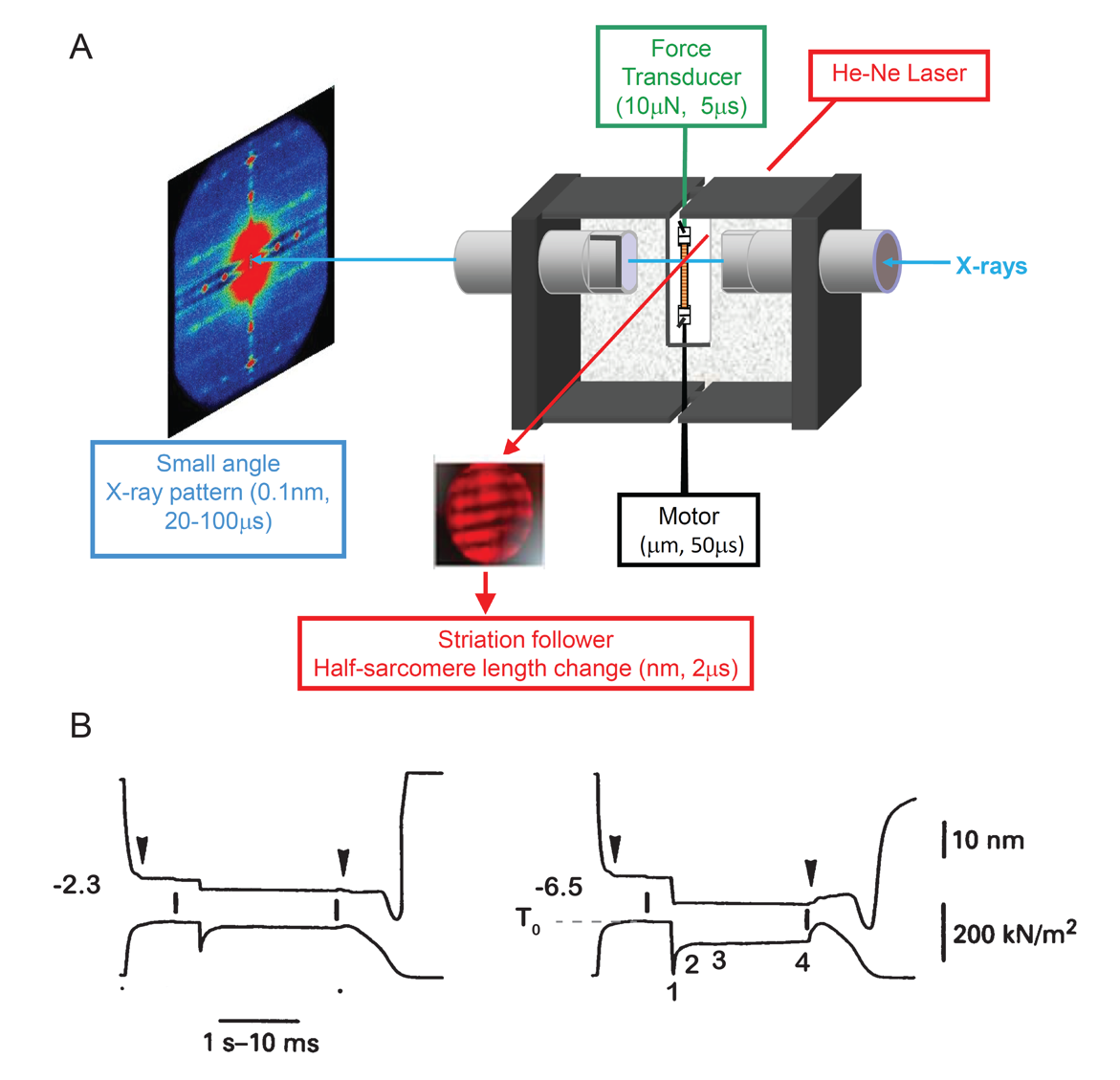 |
| --- |
| **Figure S1. Apparatus** **for high resolution half-sarcomere mechanics and X-ray measurements**. **A.** The setup. On the right: trough with the muscle fibre mounted between the levers of the loudspeaker motor (bottom) and the capacitance force transducer (top). Half-sarcomere length changes are recorded and controlled by the striation follower. On the left: diffraction pattern generated by the X-ray beam hitting the fibre. The light diffracted by sarcomeric proteins originate bright spots and lines the position and intensity of which give information about muscle structure. In bracket, figures indicate the resolution of each device. **B.** Sample records of the force transient (lower trace) elicited by step reductions in length (nm per half-sarcomere, upper trace, 2.3 nm (left) and 6.5 nm (right)), superimposed on the isometric tetanic force developed by an intact frog muscle fibre under sarcomere length clamp by a striation follower. The two dots below the records on the left mark the start and end of stimulation respectively. The portion of the records outside arrow-heads is in motor position clamp (fixed end). The portion within arrow-heads is in sarcomere length-clamp. The time base between the small bars close to the force records is 100 times faster than beyond them, in order to provide the appropriate resolution of the fast and slow events. Figures close to the force record in the right panel indicate the phases of the transient. Resting sarcomere length, 2.10 μm; temperature, 3.9 °C. Reproduced from Figure 1 of Piazzesi et al., with permission. |

The temperature of the bathing solution is continuously recorded with a thermistor during the experiment and it can be adjusted at any value between 0 °C and 40°C via a feedback system controlling the current feeding a Peltier module stuck to the aluminium plate carrying the chamber. Length changes are imposed on the muscle fibre by means of a loudspeaker coil motor able to deliver steps complete in less than 100 μs. Force changes are detected with a 50 kHz capacitance force transducer (Huxley and Lombardi . An optoelectronic device, the striation follower allows nanometer-microsecond resolution recording of length changes in a selected population of sarcomeres (range 500–1200 sarcomeres). The signal is converted into nm per half-sarcomere on the basis of the number of half sarcomere in the segment. Mechanical responses can be studied in fixed end mode (feedback signal from the position of the motor lever), sarcomere length clamp (feedback signal from the half-sarcomere length change measured by the striation follower) and force clamp (feedback signal from the force transducer). Sampling records for the first two modes are shown in Supplementary Figure 1B and in Figure 3A, for the force clamp mode in Figures 3B, 5A and 6A.

For the X-ray diffraction experiments conducted at the third-generation synchrotron (APS, Argonne, USA and ESRF, Grenoble, France) a pair of hollow and movable cylinders carrying two mica windows and the stimulating electrodes are positioned as close as possible to the sample to minimize the X-ray path in the solution. The trough is closed with a Perspex cover and sealed with silicon grease to prevent solution leakage when the plate carrying the trough is vertically mounted at the beamline, with the force transducer on the top and the motor at the bottom. Vertical mounting is required to have the sample longitudinal axis parallel to the smaller (vertical) size of the X-ray beam, to maximise the spatial resolution of X-ray signals along the meridional axis, parallel to the sample axis and record fine structure of myosin based reflections due to X-ray interference between the two arrays of motors from the two halves of each sarcomere.

**Mechanical model of the half-sarcomere**

The force generated by a muscle in isometric (fixed-end) conditions implies shortening of the contractile component (thousands of sarcomeres in series throughout the length of each muscle cell) to raise the strain in the series elastic component represented by tendons. A record of this phenomenon is given by the half-sarcomere shortening during the rise of the tetanic force in Supplementary Figure 1B (portion of the record in fixed end conditions before the first arrow-head). In length clamp conditions the changes in strain of the series elastic component that accompany the change in force are taken into account by counteracting motor movements that preserve the isometric condition of the sarcomeres. This is shown by the record within the arrow-heads in Supplementary Figure 1B: the sarcomere length clamp ensures that the quick force recovery occurs without any half-sarcomere shortening.

| 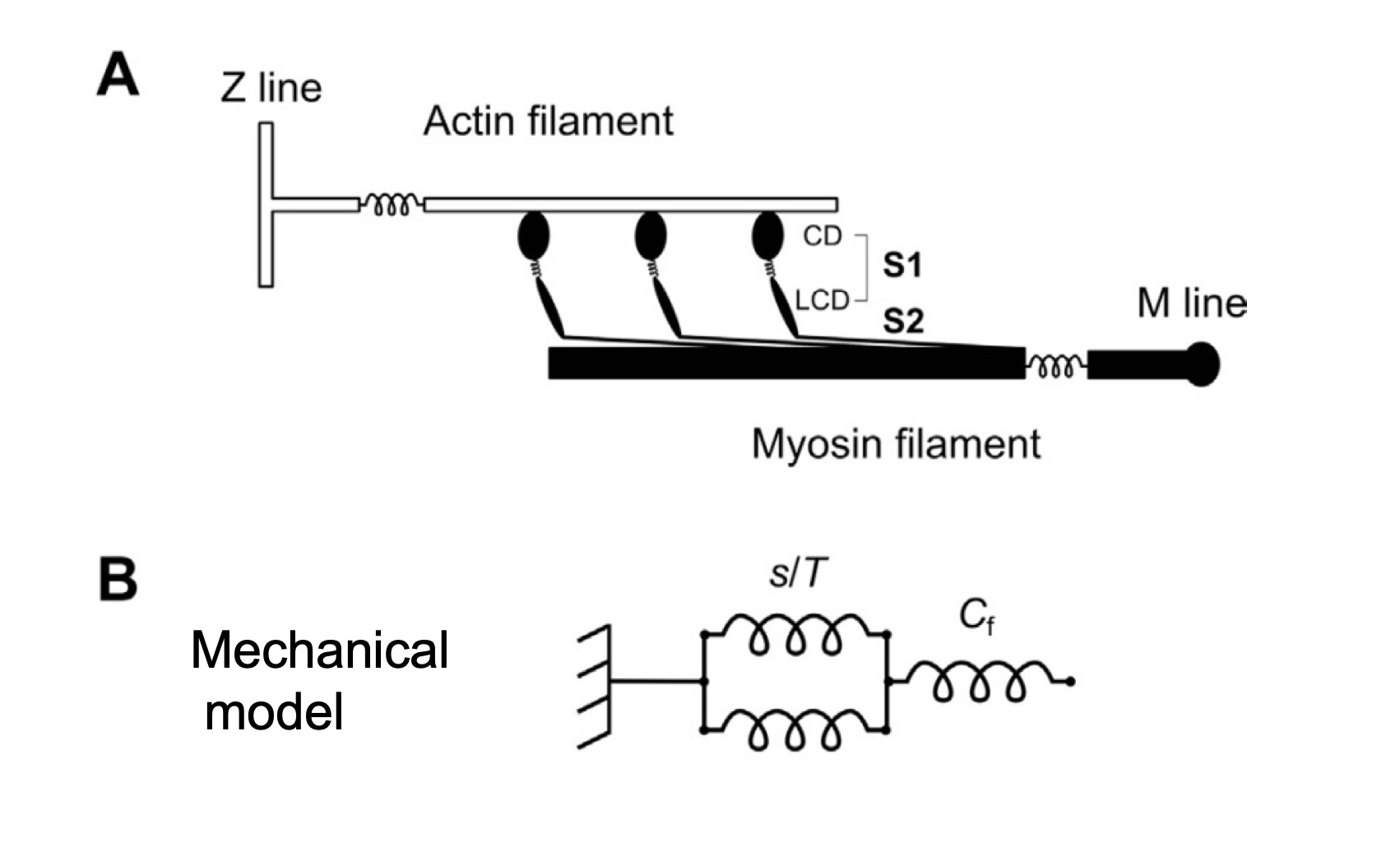 |
| --- |
| **Supplementary Figure 2. Mechanical model of the half-sarcomere. A**. Schematic representation of the half-sarcomere in which actin and myosin compliances are lumped in two springs in series with the array of myosin motors arranged in parallel. **B.** Mechanical model of the half-sarcomere: actin and myosin compliances are represented as a spring with compliance (*C*_f_) in series with the array of parallel elastic elements with a constant strain (s), representing the attached-force generating myosin motors. The compliance of the array (*s*/*T*) is inversely proportional to the level of isometric force exerted by the array that increases linearly with the number of attached motors. Reproduced from Figure 1 in Piazzesi et al. with permission. |

However, each half-sarcomere is made by a complex series-parallel network of myosin motors and myofilaments (shown in a simplified from in Supplementary Figure 2A) that can be reduced to a “linear” mechanical model, in which myosin motors are parallel force generators between the actin and myosin filaments that act as elastic elements in series. The two elastic elements can be defined by an equivalent compliance *C*_f_ in series with the array of parallel elastic elements with a constant strain (*s*), representing the attached myosin motors (Supplementary Figure 2B). The compliance of the array (*s*/*T*) is inversely proportional to the level of isometric force exerted by the array that increases linearly with the number of attached motors. Mechanical and X-ray diffraction measurements of *C*_f_ converge to a value that at *T*_0_ is ~1/2 the half-sarcomere compliance (*C*_hs_) and thus comparable to *s*/*T*_0_ . The model in Supplementary Figure 2A predicts a linear *C*_hs_ -*T* relation, in agreement with the experimental data for isometric forces above 0.2-0.3 *T*_0_. The slope of the relation estimates the value of *C*_f_ which is constant independent of *T* and the ordinate intercept estimate the strain *s* of an isometric force generating motor. At lower isometric forces, and thus lower number of in parallel force generators, the relation is progressively shifted downward with respect to that predicted by the linear model, because the reduction of number of attached motors and thus the increase in compliance of the motor array allow the presence of a parallel elastic element with a compliance two orders of magnitude higher than *s/T*_0_, titin, to emerge .

**Calculation procedures for simulations with Reaction Scheme 4**

The structurally and biochemically explicit model depicted in Figure 12 combines the kinetic scheme able to simulate the chemomechanical coupling underpinning the mechanical outputs of skinned fibres from mammalian skeletal muscle and the Huxley and Simmons model of the working stroke , which implies different conformations of the attached motors.

MATP and MADPPi-AMADPPi are detached states, AM_x_ADPPi, AM_x_ADP and AM_x_ (where *x* indicates the structural state and assumes values from 1 to 4) are states attached to an actin monomer (*A*) and A’M_x_ADPPi, A’M_x_ADP and A’M_x_ are states attached to the next monomer (*A*’) on the same actin strand shifted by 5.5 nm farther from the centre of the sarcomere. The structural change underlying the working stroke is represented by three stepwise transitions between four structural states of the attached motor (M_1_-M_2_, M_2_-M_3_, M_3_-M_4_), each responsible for an axial movement of *z* = 3.1 nm, where *z* is the axial position of the motor defined as the distance between the head-rod junction and the point of attachment of the catalytic domain on the actin filament. A linear elasticity of the myosin motor with stiffness ε = 1.2 pN nm^-1^ was assumed in the original model . Under these conditions three steps of 3.1 nm fit both the kinetic requirements necessary to simulate force and velocity transients following length and force steps respectively and the mechanical and structural constraints of a maximum sliding distance accounted for by the attached motor.

The following assumptions on the model parameters integrate those reported previously . A myosin head can attach in the AM_1_ADPPi state to one actin site (*A*) for a range of positions λ = 5.5 nm, from *x* = -2.75 to 2.75 nm (where *x* is the relative axial position between the myosin motor and *A*, and is zero for the centre of distribution of attachments of the motors in the M_1_ state). ξ is the value *x* assumes within λ (-2.75 ≤ ξ ≤ 2.75). Myosin heads are uniformly distributed along *x* so that, in isometric conditions, for each value of ξ the sum of all attached and detached heads is 1. The periodic boundary conditions imply that (*i*) in isometric conditions at the two extremities of λ there are no attached myosin motors and the sum of detached states is 1; (*ii*) sliding in the shortening direction shifts the distribution of the attached motors to negative *x* values beyond λ. When a myosin motor detaches, the time for the motor to regain the original configuration is assumed to be very short (tens of microseconds). Provided that the rate of reverse reaction at the site of detachment is low with respect to the resetting time of the myosin motor, all the motors detaching at a given *x* beyond λ regain the position, within λ, given by ξ = *x* - *n*λ, where *n* (negative for shortening) is the number of times the attached motor has exceeded one boundary in the same direction. During sliding the sum of the fractional occupancies of all states *N*(*ξ*) = 1 is given by the equation:

$$N(\xi)=MATP(\xi)+MADPPi(\xi)+M^{*}ADPPi(\xi)+\sum_{x=1}^{4} AMxADPPi(\xi+n\lambda)+\sum_{x=1}^{4} AMxADP(\xi+n\lambda)+$$

$$\sum_{x=1}^{4} AMx(\xi+n\lambda)+\sum_{x=1}^{4} A'MxADPPi(\xi+n\lambda)+\sum_{x=1}^{4} A'MxADP(\xi+n\lambda)+\sum_{x=1}^{4} A'Mx(\xi+n\lambda)=1$$

For any steady state mechanical condition (isometric contraction or steady shortening), the total flux of energy can be calculated from the flux between the states MATP and MADPPi-AMADPPi (step 2) or MADPPi-AMADPPi and AM_1._ADPPi (step 3) in the cycle. The free energy of hydrolysis of one molecule of MgATP (ΔG^ATP^) can be expressed as:

$$\Delta G^{ATP}=\Delta G_{0}+k_{B}\Theta\ln\frac{[ATP]}{[ADP][Pi]}$$

where *k_B_* (=1.381*10^-23^ JK^-1^) is the Boltzmann constant, Θ (=285.15 K) is the absolute temperature and Δ*G*_0_ is the standard free energy. Δ*G*^ATP^ is assumed to be 100 zJ in the control condition (MgATP, 5 mM; free ADP, 30 µM and free Pi, 1 mM) and it reduces by rising [Pi].

The equations expressing the *x*-dependence of the reaction rates are reported in Supplementary Table 1. The corresponding profiles are in Figure 7 of Caremani et al., 2015. The differential equations used to calculate the rates of the transition between consecutive states are reported in Supplementary Table 2. The distribution of myosin motors at any given time is calculated by numerical integration of the differential equations .

The free energy profile (G(*x*) of the various states is related to the forward k_i_(*x*) and reverse k_-i_(*x*) rate constants of the transition between neighbouring states *j* and *m* through the Gibbs equation:

$k_{i}(x)/k_{-i}(x)=\exp[(G_{j}(x)-G_{m}(x))/k_{B}\Theta$]

One rate constant of the pair is calculated from the above equation after choosing the appropriate value for the other.

In isometric conditions the time step for integration (Δt) was 25 µs. The simulation started with myosin heads distributed between MATP and AMADPPi states according to the equilibrium constant. The fraction of myosin motors in each state was calculated with an interval (Δ*x*) of 0.025 nm. Because detached myosin motors exist only within λ there are (5.5/0.025 =) 220 discrete positions for which the isometric distribution is calculated.

During the isotonic velocity transient Δt was 25 µs, while during ramp shortening Δt was chosen according to sliding velocity. The algorithm for the simulation of the isotonic condition was applied following a stepwise displacement of the isometric distribution of the attached motors by the amount corresponding to the desired drop in force. The force is calculated using the values of the rate constants at the new position *x* and compared with the desired force. If the calculated force is higher (or lower) than the desired force (within a desired range), the distribution is displaced toward negative (or positive) *x* to reduce (increase) the force. The amount of shortening (lengthening) necessary to maintain the force within the desired range originates the velocity transient and the frequency of displacement toward negative (positive) *x* the velocity of the transient. The velocity of phase 2 and phase 4 of the velocity transient are estimated, as in the experimental records, by the slope of the tangent to the initial part (phase 2) and to the steady state (phase 4) of the simulated shortening.

The force generated by an attached myosin motor at a given *x* (*F_x_*) between *x*-Δ*x*/2 and *x*+Δ*x*/2 is calculated as

$$\begin{matrix} F_{x}=\varepsilon(\sum_{i=b_{1}}^{b_{3}} xAM1_{i,x}+\sum_{i=b_{1}}^{b_{3}} (x+z)AM2_{i,x}+\sum_{i=b_{1}}^{b_{3}} (x+2z)AM3_{i,x}+\sum_{i=b_{1}}^{b_{3}} (x+3z)AM4_{i,x}+ \\ \sum_{i=b_{1}}^{b_{3}} (x+a)A'M1_{i,x}+\sum_{i=b_{1}}^{b_{3}} (x+a+z)A'M2_{i,x}+\sum_{i=b_{1}}^{b_{3}} (x+a+2z)A'M3_{i,x}+\sum_{i=b_{1}}^{b_{3}} (x+a+3z)A'M4_{i,x}) \end{matrix}$$

where *i* identifies the three biochemical states (*b*_1_=AMADPPi, *b*_2_=AMADP and *b*_3_=AM) and *a* (=5.5 nm) is the increase in strain on a motor slipping to the second actin. The average force (*F*) generated by the population of the attached myosin heads is calculated by integrating *F_x_* over *x*.

**Supplementary Table 1. Equations expressing the rate constants of the forward transitions in the reaction scheme according to the direction of the reaction flow during steady shortening.**

| *k*_1, m_, *k*_1’, m_ (M^-1^ s^-1^) | = 5.6·10^5^ |  |
| --- | --- | --- |
| *k*_2_ (s^-1^) | = 25 |  |
| *k*_3_ (s^-1^) | = 45·exp·(-0.1·(*x+x*_0_-1.5)^6^) |  |
|  |  |  |
| *k*_4, M1_ (s^-1^), *k*_4’, M1_ (s^-1^) | = 80 |  |
| *k*_4, M2_ (s^-1^), *k*_4’, M2_ (s^-1^) | = 200 |  |
| *k*_4, M3_ (s^-1^), *k*_4’, M3_ (s^-1^) | = 500 |  |
| *k*_4, M4_ (s^-1^), *k*_4’, M4_ (s^-1^) | = 1000 |  |
| *k*_5, M1_ (s^-1^), *k*_5’, M1_ (s^-1^) | = 0.5 |  |
| *k*_5, M2_ (s^-1^), *k*_5’, M2_ (s^-1^) | = 10 |  |
| *k*_5, M3_ (s^-1^), *k*_5’, M3_ (s^-1^) | =15 |  |
| *k*_5, M4_ (s^-1^) | =150+1450·exp((-3·*z*·( *x+ x*_0_+3·*z*+4))/ *k*_B_*θ*)/(1+ exp(-3·*z*·(*x+ x*_0_+3·*z* +4)/*k*_B_*θ*)) |  |
| *k*_5’, M4_ (s^-1^) | =150+1450·exp((-3·*z*·( *x+x*_0_+3·*z* +9.5))/*k*_B_*θ*)/(1+ exp(-3·*z*· (*x+ x*_0_ +3·*z* +9.5)/*k*_B_*θ*)) |  |
| *k*_6, M1_ (s^-1^) | =25+1460·exp(-*z*·*ε*·(*x +x*_0_ +1.15)/*k*_B_*θ*)/(1+ exp(-*z*·*ε*·(*x+ x*_0_+1.15)/*k*_B_*θ*) |  |
| *k*_6, M2_ (s^-1^) | = 1500·exp(-*z*·*ε*·(*x+ x*_0_-1.5+*z*)/*k*_B_*θ*)/(1+ exp(-*z*·*ε*·(*x+ x*_0_-1.5+*z*)/*k*_B_*θ*) |  |
| *k*_6, M3_ (s^-1^) | = 1500·exp(-*z*·*ε*·(*x+ x*_0_-1+2·*z*)/*k*_B_*θ*)/(1+ exp(-*z*·*ε*·(*x+ x*_0_-1+2·*z*)/*k*_B_*θ*) |  |
| *k*_6, M4_ (s^-1^) | = 1500·exp(-*z*·*ε*·(*x+ x*_0_+6+3·*z*)/*k*_B_*θ*)/(1+ exp(-*z*·*ε*·(*x+ x*_0_+6+3·*z*)/*k*_B_*θ*) |  |
| *k*_6’, M1_ (s^-1^) | =13+1460·exp(-*z*·*ε*·(*x +x*_0_ +6.65)/*k*_B_*θ*)/(1+ exp(-*z*·*ε*·(*x+ x*_0_+6.65)/*k*_B_*θ*) |  |
| *k*_6’, M2_ (s^-1^) | = 1500·exp(-*z*·*ε*·(*x+ x*_0_+4+*z*)/*k*_B_*θ*)/(1+ exp(-*z*·*ε*·(*x+ x*_0_+4+*z*)/*k*_B_*θ*) |  |
| *k*_6’, M3_ (s^-1^) | = 1500·exp(-*z*·*ε*·(*x+ x*_0_+4.5+2·*z*)/*k*_B_*θ*)/(1+ exp(-*z*·*ε*·(*x+ x*_0_+4.5+2·*z*)/*k*_B_*θ*) |  |
| *k*_6’, M4_ (s^-1^) | = 1500·exp(-*z*·*ε*·(*x+ x*_0_+11.5+3·*z*)/*k*_B_*θ*)/(1+ exp(-*z*·*ε*·(*x+ x*_0_+11.5+3·*z*)/*k*_B_*θ*) |  |
| k_7_ (s^-1^), k_7’_ (s^-1^) | = 2000 |  |
| *k*_8, M2_ (s^-1^) | = 0.1·exp(-*z*·*ε*·(*x+ x*_0_+0.5+z)/*k*_B_*θ*)/(1+ exp(-*z*·*ε*·(*x+ x*_0_+0.5+z)/*k*_B_*θ*)  = 0 | *x* < -1  *x* ≥ -1 |
| *k*_8, M3_ (s^-1^) | = 60·exp(-*z*·*ε*·(*x+ x*_0_-1+2·z)/*k*_B_*θ*)/(1+ exp(-*z*·*ε*·(*x+ x*_0_-1+2·z)/*k*_B_*θ*)  = 0 | *x* < -1  *x* ≥ -1 |
| *k*_8, M4_ (s^-1^) | = 0.1·exp(-*z*·*ε*·(*x+ x*_0_-1.3+3·z)/*k*_B_*θ*)/(1+ exp(-*z*·*ε*·(*x+ x*_0_-1.3+3·z)/*k*_B_*θ*)  = 0 | *x* < -1  *x* ≥ -1 |
| k_9, M2_ (s^-1^) | = 1000·exp(-*z*·*ε*·(*x+ x*_0_+0.5+z)/*k*_B_*θ*)/(1+ exp(-*z*·*ε*·(*x+ x*_0_+0.5+z)/*k*_B_*θ*)  = 0 | *x* < -1  *x* ≥ -1 |
| k_9, M3_ (s^-1^) | = 1200·exp(-*z*·*ε*·(*x+ x*_0_-1+2·z)/*k*_B_*θ*)/(1+ exp(-*z*·*ε*·(*x+ x*_0_-1+2·z)/*k*_B_*θ*)  = 0 | *x* < -1  *x* ≥ -1 |
| k_9, M4_ (s^-1^) | = 10·exp(-*z*·*ε*·(*x+ x*_0_-1.3+3·z)/*k*_B_*θ*)/(1+ exp(-*z*·*ε*·(*x+ x*_0_-1.3+3·z)/*k*_B_*θ*)  = 0 | *x* < -1  *x* ≥ -1 |
| *k*_w1_ (s^-1^) | = 73500·exp(-*z*·*ε*·(*x+ x*_0_+0.5)/*k*_B_*θ*)/(1+ exp(-*z*·*ε*·(*x+ x*_0_+0.5)/*k*_B_*θ*) |  |
| *k*_w2_ (s^-1^) | = 49000·exp(-*z*·*ε*·(*x+ x*_0_+0.5+*z*)/*k*_B_*θ*)/(1+ exp(-*z*·*ε*·(*x+ x*_0_+0.5+*z*)/*k*_B_*θ*) |  |
| *k*_w3_ (s^-1^) | = 24500·exp(-*z*·*ε*·(*x+ x*_0_+0.5+2·*z*)/*k*_B_*θ*)/(1+ exp(-*z*·*ε*·(*x+ x*_0_+0.5+2·*z*)/*k*_B_*θ*) |  |
| *k*_w1’_ (s^-1^) | = 73500·exp(-*z*·*ε*·(*x+ x*_0_+6)/*k*_B_*θ*)/(1+ exp(-*z*·*ε*·(*x+ x*_0_+6)/*k*_B_*θ*) |  |
| *k*_w2’_ (s^-1^) | = 49000·exp(-*z*·*ε*·(*x+ x*_0_+6+*z*)/*k*_B_*θ*)/(1+ exp(-*z*·*ε*·(*x+ x*_0_+6+*z*)/*k*_B_*θ*) |  |
| *k*_w3’_ (s^-1^) | = 24500·exp(-*z*·*ε*·(*x+ x*_0_+6+2·*z*)/*k*_B_*θ*)/(1+ exp(-*z*·*ε*·(*x+ x*_0_+6+2·*z*)/*k*_B_*θ*) |  |

The rate constants of the transitions between different biochemical states or between different actin monomers are indicated as *k*_lm_ where *l* (from 1 to 9) identifies the transition according to the scheme of Figure 12 and *m* (M_1_→M_4_) indicates the structural state involved in the transition. The rate constants of the structural transitions for the same biochemical state are indicated as *k*_w1_, *k*_w2_ and *k*_w3_ for the transitions M_1_→M_2_, M_2_→M_3_ and M_3_→M_4_, respectively. These ‘structural’ rate constants have the same *x*-dependence for the different biochemical states but their values are multiplied by 0.01 for AM and A_M states. *x*_0_ is the strain of the M_1_ state at *x* = 0, which has been set to 1.15 nm. The prime symbol (‘) is added to the subscript if the transition occurs on the second actin. Modified from .

**Supplementary Table 2 Differential equations used to calculate the rates of the transition between consecutive states.**

| *δ*MATP(*x*, *t*)/*δt* = *k*_-2_(*x*)AMADPPi(*x*, *t*) + *k*_1,M1_(*x*)AM_1_(*x*, *t*) + *k*_1,M2_(*x*)AM_2_(*x*, *t*) + *k*_1,M3_(*x*)AM_3_(*x*, *t*) + *k*_1,M4_(*x*)AM_4_(*x*, *t*) + *k*_1’,M1_(*x*)A’M_1_(*x*, *t*) + *k*_1’,M2_(*x*)A’M_2_(*x*, *t*) + *k*_1’,M3_(*x*)A’M_3_(*x*, *t*) + *k*_1’,M4_(*x*)A’M_4_(*x*, *t*) + *k*_7_(*x*)M^*^ADPPi(*x*, *t*) - (*k*_2_(*x*) + *k*_-1,M1_(*x*) + *k*_-1,M2_(*x*) + *k*_-1,M3_(*x*) + *k*_-1,M4_(*x*) + *k*_-1’,M1_(*x*) + *k*_-1’,M2_(*x*) + *k*_-1’,M3_(*x*) + *k*_-1’,M4_(*x*) + *k*_-7_(*x*)) ∙MATP(*x*, *t*) - *v*(*δ*MATP(*x*, *t*)/*δx*); |
| --- |
| *δ*AMADPPi(*x*, *t*)/*δt* = *k*_2_(*x*)MATP(*x*, *t*) + *k*_-3_(*x*)AM_1_ADPPi(*x*, *t*) - (*k*_-2_(*x*) + *k*_3_(*x*)) ∙AMADPPi(*x*, *t*) - *v*(*δ*AMADPPi(*x*, *t*)/*δx*); |
| *δ*AM_1_ADPPi(*x*, *t*)/*δt* = *k*_3_(*x*)AMADPPi(*x*, *t*) + *k*_-4,M1_(*x*)AM_1_ADP(*x*, *t*) + *k*_-1w_(*x*)AM_2_ADPPi(*x*, *t*) + *k*_-6,M1_(*x*)M^*^ADPPi(*x*, *t*) - (*k*_-3_(*x*) + *k*_4,M1_(*x*) + *k*_1w_(*x*) + *k*_6,M1_(*x*)) ∙AM_1_ADPPi (*x*, *t*) - *v*(*δ*AM_1_ADPPi (*x*, *t*)/*δx*); |
| *δ*AM_2_ADPPi(*x*, *t*)/*δt* = *k*_1w_(*x*)AM_1_ADPPi(*x*, *t*) + *k*_-4,M2_(*x*)AM_2_ADP(*x*, *t*) + *k*_-2w_(*x*)AM_3_ADPPi(*x*, *t*) + *k*_-6,M2_(*x*)M^*^ADPPi(*x*, *t*) + *k*_-8,M2_(*x*)A’M_2_ADPPi(*x*, *t*) - (*k*_-1w_(*x*) + *k*_4,M2_(*x*) + *k*_2w_(*x*) + *k*_8,M2_(*x*) + *k*_6,M2_(*x*)) ∙AM_2_ADPPi (*x*, *t*) - *v*(*δ*AM_2_ADPPi (*x*, *t*)/*δx*); |
| *δ*AM_3_ADPPi(*x*, *t*)/*δt* = *k*_2w_(*x*)AM_2_ADPPi(*x*, *t*) + *k*_-4,M3_(*x*)AM_3_ADP(*x*, *t*) + *k*_-3w_(*x*)AM_4_ADPPi(*x*, *t*) + *k*_-6,M3_(*x*)M^*^ADPPi(*x*, *t*) + *k*_-8,M3_(*x*)A’M_3_ADPPi(*x*, *t*) - (*k*_-2w_(*x*) + *k*_4,M3_(*x*) + *k*_3w_(*x*) + *k*_6,M3_(*x*) + *k*_8,M3_(*x*)) ∙AM_3_ADPPi (*x*, *t*) - *v*(*δ*AM_3_ADPPi (*x*, *t*)/*δx*); |
| *δ*AM_4_ADPPi(*x*, *t*)/*δt* = *k*_3w_(*x*)AM_3_ADPPi(*x*, *t*) + *k*_-4,M4_(*x*)AM_4_ADP(*x*, *t*) + *k*_-6,M4_(*x*)M^*^ADPPi(*x*, *t*) - (*k*_-3w_(*x*) + *k*_4,M4_(*x*) + *k*_6,M4_(*x*)) ∙AM_4_ADPPi (*x*, *t*) - *v*(*δ*AM_4_ADPPi (*x*, *t*)/*δx*); |
| *δ*AM_1_ADP(*x*, *t*)/*δt* = *k*_4,M1_(*x*)AM_1_ADPPi(*x*, *t*) + *k*_-5,M1_(*x*)AM_1_(*x*, *t*) + *k*_-1w_(*x*)AM_2_ADP(*x*, *t*) - (*k*_-4,M1_(*x*) + *k*_5,M1_(*x*) + *k*_1w_(*x*)) ∙AM_1_ADP(*x*, *t*) - *v*(*δ*AM_1_ADP (*x*, *t*)/*δx*); |
| *δ*AM_2_ADP(*x*, *t*)/*δt* = *k*_1w_(*x*)AM_1_ADP(*x*, *t*) + *k*_-5,M2_(*x*)AM_2_(*x*, *t*) + *k*_-2w_(*x*)AM_3_ADP(*x*, *t*) + *k*_-9,M2_(*x*)A’M_2_ADP(*x*, *t*) + *k*_4,M2_(*x*)AM_2_ADPPi(*x*, *t*) - (*k*_-1w_(*x*) + *k*_5,M2_(*x*) + *k*_2w_(*x*) + *k*_9,M2_(*x*) + *k*_-4,M2_(*x*)) ∙AM_2_ADP(*x*, *t*) - *v*(*δ*AM_2_ADP (*x*, *t*)/*δx*); |
| *δ*AM_3_ADP(*x*, *t*)/*δt* = *k*_2w_(*x*)AM_2_ADP(*x*, *t*) + *k*_-5,M3_(*x*)AM_3_(*x*, *t*) + *k*_-3w_(*x*)AM_4_ADP(*x*, *t*) + *k*_4,M3_(*x*) AM_3_ADPPi(*x*, *t*) + *k*_-9,M3_(*x*)A’M_3_ADP(*x*, *t*) - (*k*_-2w_(*x*) + *k*_5,M3_(*x*) + *k*_3w_(*x*) + *k*_-4,M3_(*x*) + *k*_9,M3_(*x*)) ∙AM_3_ADP(*x*, *t*) - *v*(*δ*AM_3_ADP (*x*, *t*)/*δx*); |
| *δ*AM_4_ADP(*x*, *t*)/*δt* = *k*_3w_(*x*)AM_3_ADP(*x*, *t*) + *k*_-5,M4_(*x*)AM_4_(*x*, *t*) + *k*_4,M4_(*x*) AM_4_ADPPi(*x*, *t*) - (*k*_-3w_(*x*) + *k*_5,M4_(*x*) + *k*_-4,M4_(*x*))∙AM_4_ADP(*x*, *t*) - *v*(*δ*AM_4_ADP (*x*, *t*)/*δx*); |
| *δ*AM_1_(*x*, *t*)/*δt* = *k*_5,M1_(*x*)AM_1_ADP(*x*, *t*) + *k*_-1,M1_(*x*)MATP(*x*, *t*) + *k*_-1w_(*x*)AM_2_(*x*, *t*) - (*k*_-5,M1_(*x*) + *k*_1,M1_(*x*) + *k*_1w_(*x*)) ∙AM_1_(*x*, *t*) - *v*(*δ*AM_1_ (*x*, *t*)/*δx*); |
| *δ*AM_2_(*x*, *t*)/*δt* = *k*_5,M2_(*x*)AM_2_ADP(*x*, *t*) + *k*_-1,M2_(*x*)MATP(*x*, *t*) + *k*_1w_(*x*)AM_1_(*x*, *t*) + *k*_-2w_(*x*)AM_3_(*x*, *t*) - (*k*_-5,M2_(*x*) + *k*_1,M2_(*x*) + *k*_-1w_(*x*) + *k*_2w_(*x*)) ∙AM_2_(*x*, *t*) - *v*(*δ*AM_2_ (*x*, *t*)/*δx*); |
| *δ*AM_3_(*x*, *t*)/*δt* = *k*_5,M3_(*x*)AM_3_ADP(*x*, *t*) + *k*_-1,M3_(*x*)MATP(*x*, *t*) + *k*_2w_(*x*)AM_2_(*x*, *t*) + *k*_-3w_(*x*)AM_4_(*x*, *t*) - (*k*_-5,M3_(*x*) + *k*_1,M3_(*x*) + *k*_-2w_(*x*) + *k*_3w_(*x*)) ∙AM_3_(*x*, *t*) - *v*(*δ*AM_3_ (*x*, *t*)/*δx*); |
| *δ*AM_4_(*x*, *t*)/*δt* = *k*_5,M4_(*x*)AM_4_ADP(*x*, *t*) + *k*_-1,M4_(*x*)MATP(*x*, *t*) + *k*_3w_(*x*)AM_3_(*x*, *t*) - (*k*_-5,M4_(*x*) + *k*_1,M4_(*x*) + *k*_-3w_(*x*)) ∙AM_4_(*x*, *t*) - *v*(*δ*AM_4_ (*x*, *t*)/*δx*);  *δ*M^*^ADPPi(*x*, *t*)/*δt* = *k*_6,M1_(*x*)AM_1_ADPPi(*x*, *t*) + *k*_6,M2_(*x*)AM_2_ADPPi(*x*, *t*) + *k*_6,M3_(*x*)AM_3_ADPPi(*x*, *t*) + *k*_6,M4_(*x*)AM_4_ADPPi(*x*, *t*) + *k*_-7_(*x*)MATP(*x*, *t*) - (*k*_-6,M1_(*x*) + *k*_-6,M2_(*x*) + *k*_-6,M3_(*x*) + *k*_-6,M4_(*x*) + *k*_7_(*x*)) ∙M^*^ADPPi (*x*, *t*) - *v*(*δ*M^*^ADPPi (*x*, *t*)/*δx*); |
| *δ*A’M_1_ADPPi(*x*, *t*)/*δt* = *k*_-4’,M1_(*x*)A’M_1_ADP(*x*, *t*) + *k*_-1w_(*x*)A’M_2_ADPPi(*x*, *t*) - (*k*_4’,M1_(*x*) + *k*_1w_(*x*)) ∙A’M_1_ADPPi (*x*, *t*) - *v*(*δ*A’M_1_ADPPi (*x*, *t*)/*δx*); |
| *δ*A’M_2_ADPPi(*x*, *t*)/*δt* = *k*_-4’,M2_(*x*)A’M_2_ADP(*x*, *t*) + *k*_1w_(*x*)A’M_1_ADPPi(*x*, *t*) + *k*_-2w_(*x*)A’M_3_ADPPi(*x*, *t*) + *k*_8,M2_(*x*)AM_2_ADPPi(*x*, *t*) - (*k*_4’,M2_(*x*) + *k*_-1w_(*x*) + *k*_2w_(*x*) + *k*_-8,M2_(*x*))∙A’M_2_ADPPi (*x*, *t*) - *v*(*δ*A’M_2_ADPPi (*x*, *t*)/*δx*); |
| *δ*A’M_3_ADPPi(*x*, *t*)/*δt* = *k*_-4’,M3_(*x*)A’M_3_ADP(*x*, *t*) + *k*_2w_(*x*)A’M_2_ADPPi(*x*, *t*) + *k*_-3w_(*x*)A’M_4_ADPPi(*x*, *t*) + *k*_8,M3_(*x*)AM_3_ADPPi(*x*, *t*) - (*k*_4’,M3_(*x*) + *k*_-2w_(*x*) + *k*_3w_(*x*) + *k*_-8,M3_(*x*))∙A’M_3_ADPPi (*x*, *t*) - *v*(*δ*A’M_3_ADPPi (*x*, *t*)/*δx*); |
| *δ*A’M_4_ADPPi(*x*, *t*)/*δt* = *k*_-4’,M4_(*x*)A’M_4_ADP(*x*, *t*) + *k*_3w_(*x*)A’M_3_ADPPi(*x*, *t*) - (*k*_4’,M4_(*x*) + *k*_-3w_(*x*))∙A’M_4_ADPPi (*x*, *t*) - *v*(*δ*A’M_4_ADPPi (*x*, *t*)/*δx*); |
| *δ*A’M_1_ADP(*x*, *t*)/*δt* = *k*_-5’,M1_(*x*)A’M_1_(*x*, *t*) + *k*_-1w_(*x*)A’M_2_ADP(*x*, *t*) + *k*_4’,M1_(*x*)A’M_1_ADPPi(*x*, *t*) - (*k*_5’,M1_(*x*) + *k*_1w_(*x*) + *k*_-4’,M1_(*x*))∙A’M_1_ADP (*x*, *t*) - *v*(*δ*A’M_1_ADP (*x*, *t*)/*δx*); |
| *δ*A’M_2_ADP(*x*, *t*)/*δt* = *k*_-5’,M2_(*x*)A’M_2_(*x*, *t*) + *k*_1w_(*x*)A’M_1_ADP(*x*, *t*) + *k*_4’,M2_(*x*)A’M_2_ADPPi(*x*, *t*) + *k*_-2w_(*x*)A’M_3_ADP(*x*, *t*) + *k*_9,M2_(*x*)AM_2_ADP(*x*, *t*) - (*k*_5’,M2_(*x*) + *k*_-1w_(*x*) + *k*_-4’,M2_(*x*) + *k*_2w_(*x*) + *k*_-9,M2_(*x*))∙A’M_2_ADP (*x*, *t*) - *v*(*δ*A’M_2_ADP (*x*, *t*)/*δx*); |
| *δ*A’M_3_ADP(*x*, *t*)/*δt* = *k*_-5’,M3_(*x*)A’M_3_(*x*, *t*) + *k*_2w_(*x*)A’M_2_ADP(*x*, *t*) + *k*_4’,M3_(*x*)A’M_3_ADPPi(*x*, *t*) + *k*_-3w_(*x*)A’M_4_ADP(*x*, *t*) + *k*_9,M3_(*x*)AM_3_ADP(*x*, *t*) - (*k*_5’,M3_(*x*) + *k*_-2w_(*x*) + *k*_-4’,M3_(*x*) + *k*_3w_(*x*) + *k*_-9,M3_(*x*))∙A’M_3_ADP (*x*, *t*) - *v*(*δ*A’M_3_ADP (*x*, *t*)/*δx*); |
| *δ*A’M_4_ADP(*x*, *t*)/*δt* = *k*_-5’,M4_(*x*)A’M_4_(*x*, *t*) + *k*_3w_(*x*)A’M_3_ADP(*x*, *t*) + *k*_4’,M4_(*x*)A’M_4_ADPPi(*x*, *t*) - (*k*_5’,M4_(*x*) + *k*_-3w_(*x*) + *k*_-4’,M4_(*x*))∙A’M_4_ADP (*x*, *t*) - *v*(*δ*A’M_4_ADP (*x*, *t*)/*δx*); |
| *δ*A’M_1_(*x*, *t*)/*δt* = *k*_5’,M1_(*x*) A’M_1_ADP(*x*, *t*) + *k*_-1’,M1_(*x*)MATP(*x*, *t*) + *k*_-1w_(*x*) A’M_2_(*x*, *t*) - (*k*_-5’,M1_(*x*) + *k*_1’,M1_(*x*) + *k*_1w_(*x*)) ∙ A’M_1_(*x*, *t*) - *v*(*δ*A’M_1_ (*x*, *t*)/*δx*); |
| *δ* A’M_2_(*x*, *t*)/*δt* = *k*_5’,M2_(*x*) A’M_2_ADP(*x*, *t*) + *k*_-1’,M2_(*x*)MATP(*x*, *t*) + *k*_1w_(*x*) A’M_1_(*x*, *t*) + *k*_-2w_(*x*) A’M_3_(*x*, *t*) - (*k*_-5’,M2_(*x*) + *k*_1’,M2_(*x*) + *k*_-1w_(*x*) + *k*_2w_(*x*)) ∙ A’M_2_(*x*, *t*) - *v*(*δ*A’M_2_ (*x*, *t*)/*δx*); |
| *δ* A’M_3_(*x*, *t*)/*δt* = *k*_5’,M3_(*x*) A’M_3_ADP(*x*, *t*) + *k*_-1’,M3_(*x*)MATP(*x*, *t*) + *k*_2w_(*x*) A’M_2_(*x*, *t*) + *k*_-3w_(*x*) A’M_4_(*x*, *t*) - (*k*_-5’,M3_(*x*) + *k*_1’,M3_(*x*) + *k*_-2w_(*x*) + *k*_3w_(*x*)) ∙ A’M_3_(*x*, *t*) - *v*(*δ*A’M_3_ (*x*, *t*)/*δx*); |
| *δ* A’M_4_(*x*, *t*)/*δt* = *k*_5’,M4_(*x*) A’M_4_ADP(*x*, *t*) + *k*_-1’,M4_(*x*)MATP(*x*, *t*) + *k*_3w_(*x*) A’M_3_(*x*, *t*) - (*k*_-5’,M4_(*x*) + *k*_1’,M4_(*x*) + *k*_-3w_(*x*)) ∙ A’M_4_(*x*, *t*) - *v*(*δ*A’M_4_ (*x*, *t*)/*δx*); |

References

Barclay, C.J., Woledge, R.C., and Curtin, N.A. (2010). Inferring crossbridge properties from skeletal muscle energetics. *Prog Biophys Mol Biol* 102(1)**,** 53-71. doi: 10.1016/j.pbiomolbio.2009.10.003.

Brunello, E., Caremani, M., Melli, L., Linari, M., Fernandez-Martinez, M., Narayanan, T., et al. (2014). The contributions of filaments and cross-bridges to sarcomere compliance in skeletal muscle. *J Physiol* 592(17)**,** 3881-3899. doi: 10.1113/jphysiol.2014.276196.

Caremani, M., Melli, L., Dolfi, M., Lombardi, V., and Linari, M. (2013). The working stroke of the myosin II motor in muscle is not tightly coupled to release of orthophosphate from its active site. *J Physiol* 591(20)**,** 5187-5205. doi: 10.1113/jphysiol.2013.257410.

Caremani, M., Melli, L., Dolfi, M., Lombardi, V., and Linari, M. (2015). Force and number of myosin motors during muscle shortening and the coupling with the release of the ATP hydrolysis products. *J Physiol* 593(15)**,** 3313-3332. doi: 10.1113/JP270265.

Dobbie, I., Linari, M., Piazzesi, G., Reconditi, M., Koubassova, N., Ferenczi, M.A., et al. (1998). Elastic bending and active tilting of myosin heads during muscle contraction. *Nature* 396(6709)**,** 383-387. doi: 10.1038/24647.

Fusi, L., Brunello, E., Reconditi, M., Piazzesi, G., and Lombardi, V. (2014). The non-linear elasticity of the muscle sarcomere and the compliance of myosin motors. *J Physiol* 592(5)**,** 1109-1118. doi: 10.1113/jphysiol.2013.265983.

Huxley, A.F., and Lombardi, V. (1980). A sensitive force transducer with resonant frequency 50 kHz. *J Physiol* 305**,** 15-16P.

Huxley, A.F., Lombardi, V., and Peachey, L.D. (1981). A system for fast recording of longitudinal displacement of a striated muscle fibre. *J Physiol* 317**,** 12P-13P.

Huxley, A.F., and Simmons, R.M. (1971). Proposed mechanism of force generation in striated muscle. *Nature* 233(5321)**,** 533-538.

Huxley, H.E., Stewart, A., Sosa, H., and Irving, T. (1994). X-ray diffraction measurements of the extensibility of actin and myosin filaments in contracting muscle. *Biophys J* 67(6)**,** 2411-2421.

Linari, M., Aiazzi, A., Dolfi, M., Piazzesi, G., and Lombardi, V. (1993). A system for studying tension transients in segments of skinned muscle fibres from rabbit psoas. *J Physiol* 473**,** 8P.

Linari, M., Caremani, M., Piperio, C., Brandt, P., and Lombardi, V. (2007). Stiffness and fraction of Myosin motors responsible for active force in permeabilized muscle fibers from rabbit psoas. *Biophys J* 92(7)**,** 2476-2490. doi: 10.1529/biophysj.106.099549.

Lombardi, V., Piazzesi, G., and Goldman, Y.E. (1990). Following tetanic stimulation, relaxation is accelerated by quick stretches of frog single muscle fibres. *J Physiol* 426**,** 38P.

Piazzesi, G., Dolfi, M., Brunello, E., Fusi, L., Reconditi, M., Bianco, P., et al. (2014). The myofilament elasticity and its effect on kinetics of force generation by the myosin motor. *Arch Biochem Biophys* 552-553**,** 108-116. doi: 10.1016/j.abb.2014.02.017.

Piazzesi, G., Francini, F., Linari, M., and Lombardi, V. (1992). Tension transients during steady lengthening of tetanized muscle fibres of the frog. *J Physiol* 445**,** 659-711.

Piazzesi, G., and Lombardi, V. (1995). A cross-bridge model that is able to explain mechanical and energetic properties of shortening muscle. *Biophys J* 68(5)**,** 1966-1979.

Piazzesi, G., Reconditi, M., Linari, M., Lucii, L., Sun, Y.B., Narayanan, T., et al. (2002). Mechanism of force generation by myosin heads in skeletal muscle. *Nature* 415(6872)**,** 659-662. doi: 10.1038/415659a.

Powers, J.D., Bianco, P., Pertici, I., Reconditi, M., Lombardi, V., and Piazzesi, G. (2020). Contracting striated muscle has a dynamic I-band spring with an undamped stiffness 100 times larger than the passive stiffness. *J Physiol* 598(2)**,** 331-345. doi: 10.1113/JP278713.

Reconditi, M., Linari, M., Lucii, L., Stewart, A., Sun, Y.B., Boesecke, P., et al. (2004). The myosin motor in muscle generates a smaller and slower working stroke at higher load. *Nature* 428(6982)**,** 578-581. doi: 10.1038/nature02380

Wakabayashi, K., Sugimoto, Y., Tanaka, H., Ueno, Y., Takezawa, Y., and Amemiya, Y. (1994). X-ray diffraction evidence for the extensibility of actin and myosin filaments during muscle contraction. *Biophys J* 67(6)**,** 2422-2435.
